# P2X_7_ Receptors in Neurodegeneration: Potential Therapeutic Applications From Basic to Clinical Approaches

**DOI:** 10.3389/fncel.2021.617036

**Published:** 2021-04-06

**Authors:** Paul R. Territo, Hamideh Zarrinmayeh

**Affiliations:** ^1^Department of Medicine, Indiana University School of Medicine, Indianapolis, IN, United States; ^2^Department of Radiology and Imaging Sciences, Indiana University School of Medicine, Indianapolis, IN, United States

**Keywords:** purinergic signaling, neurodegeneration, neuroinflammation, neuropsychiatric disease, P2X7R

## Abstract

Purinergic receptors play important roles in central nervous system (CNS), where the bulk of these receptors are implicated in neuroinflammatory responses and regulation of cellular function of neurons, microglial and astrocytes. Within the P2X receptor family, P2X_7_ receptor is generally known for its inactivity in normal conditions and activation by moderately high concentrations (>100 μM) of extracellular adenosine 5′-triphosphate (ATP) released from injured cells as a result of brain injury or pathological conditions. Activation of P2X_7_R contributes to the activation and proliferation of microglia and directly contribute to neurodegeneration by provoking microglia-mediated neuronal death, glutamate-mediated excitotoxicity, and NLRP3 inflammasome activation that results in initiation, maturity and release of the pro-inflammatory cytokines and generation of reactive oxygen and nitrogen species. These components of the inflammatory response play important roles in many neural pathologies and neurodegeneration disorders. In CNS, expression of P2X_7_R on microglia, astrocytes, and oligodendrocytes are upregulated under neuroinflammatory conditions. Several *in vivo* studies have demonstrated beneficial effects of the P2X_7_ receptor antagonists in animal model systems of neurodegenerative diseases. A number of specific and selective P2X_7_ receptor antagonists have been developed, but only few of them have shown efficient brain permeability. Finding potent and selective P2X_7_ receptor inhibitors which are also CNS penetrable and display acceptable pharmacokinetics (PK) has presented challenges for both academic researchers and pharmaceutical companies. In this review, we discuss the role of P2X_7_ receptor function in neurodegenerative diseases, the pharmacological inhibition of the receptor, and PET radiopharmaceuticals which permit non-invasive monitoring of the P2X_7_ receptor contribution to neuroinflammation associated with neurodegeneration.

## Introduction

Purinergic receptors or purinoceptors are plasma membrane proteins found in almost all mammalian tissues including the central nervous system (CNS) (Tozaki-Saitoh et al., 2011). These receptors participate in the synaptic processes via communications between neuron-glia and glia with other glia cell types (i.e., astrocytes, oligodendrocytes, and microglia) (Tozaki-Saitoh et al., 2011). Based on their endogenous ligands, the purinergic receptors are classified into P1 and P2 categories (Burnstock, 2008). P1 or adenosine receptors are a family of G protein-coupled receptors (GPCR) with four subtypes: A_1_, A_2A_, A_2B_, and A_3_. P2 receptors are further divided into the two structurally and functionally unique families of receptors that mediate intracellular signaling evoked by extracellular ATP. The ligand-gated ion channel P2X receptors with seven subtypes: P2X_1−7_ and the G protein-coupled metabotropic P2Y receptors with eight subtypes: P2Y_1, 2, 4, 6, 11, 12, 13, 14_ (Ralevic and Burnstock, 1998; Burnstock, 2018). Several members of the purinergic receptors, specifically play major roles in CNS disorders including: Alzheimer's disease (AD), Parkinson disease (PD), Huntington's disease (HD), Frontotemporal dementia (FD), Amyotrophic Lateral Sclerosis (ALS), Multiple Scleroses (MS), Traumatic Brain Injury (TBI), stroke, cerebral ischemia, epilepsy, psychiatric diseases, sleep disorder, and neuropathic pain (Burnstock, 2008, 2016; Tozaki-Saitoh et al., 2011; Beamer et al., 2016).

Depending on the receptor subtype, adenosine and the P2Y receptors are coupled to Gq/Gi/Gs proteins (Puchalowicz et al., 2015). Gq proteins activation prompt a signaling cascade through phospholipase C/inositol-1,4,5-triphosphate (PLC/IP3), a process that promotes the release of Ca^2+^ from the endoplasmic reticulum into the cytoplasm. Activation of the Gs/Gi proteins effect stimulation or inhibition of adenylate cyclase that subsequently modifies the production of cyclic AMP (cAMP), while the non-selective P2X ion channel receptors facilitate cellular exchange of cations like Ca^2+^, Mg^2+^, Na^+^, and K^+^ (Surprenant and North, 2009; Puchalowicz et al., 2015).

The energy source for neurons and glial cells, Adenosine 5′-triphosphate (ATP), also acts as an extracellular purinergic signaling that controls communication between brain cells (Burnstock, 2006a). The steady concentration of cytosolic ATP ranges between 5 to 10 mM, but very low nM in the extracellular space (Bhattacharya and Biber, 2016). However, under pathological conditions, and during CNS insults, high concentration of ATP is released from the damaged cells to the extracellular space as a danger signal and alters calcium signaling through modulation of purinergic receptors, and in turn results in neuroinflammatory responses, excitotoxicity, and apoptosis, a cascade of events that eventually damages the neurons (Yegutkin, 2008; Roszek and Czarnecka, 2015).

High level of extracellular ATP signals microglia to undergo chemotaxis to the site of injury in order to remove cell debris from these sites (Inoue, 2008). Microglial activation (Domercq et al., 2013) result in upregulation of P2X_4_ and P2X_7_ (Di Virgilio et al., 2017) and downregulation of P2Y_12_ receptors expression (Haynes et al., 2006). The fine balance between expressions of these three receptors dictates the destiny of microglia (Vazquez-Villoldo et al., 2014). A relatively high expression levels of P2X_4_ and P2X_7_ receptors are indicator of pro-inflammatory M1 phenotype microglia activation (Burnstock, 2007). Additionally, release of the large quantity of ATP [hundreds of micromolar (μM)], activates P2Y_1_ receptor that enables movement of ramified microglia to the damage site, while P2Y_6_ receptor, a normally expressed receptor on the activated microglia, initiates phagocytosis process (Burnstock, 2008; Boue-Grabot and Pankratov, 2017). Moreover, conversion of extracellular ATP to adenosine takes place by ectonucleotidases CD39 and CD73 that are present in microglia (Braun et al., 2000) and in turn activates adenosine receptors (Fredholm et al., 2001; Choi et al., 2015). Both adenosine and ATP are essential modulators of neuroinflammatory responses, excitotoxicity, oxidative stress and cell death, especially via A_2A_ and P2X_7_ receptors activity, respectively (Cunha, 2016; Faas et al., 2017; He et al., 2017; Vuorimaa et al., 2017).

The ligand-gated ion channel P2X receptors have seven subunits that vary in length ranging from 377 amino acid residues in the P2X_6_ receptor to 595 residues in the P2X_7_ receptor (North, 2002). P2X_7_R is mostly coexpressed with P2X_4_R, and the two receptors are proven not to form heteromeric assemblies and function only as homomers (Guo et al., 2007). However, P2X_7_R homotrimers were able to co-immunoprecipitate with P2X_4_ and as such, there is an evidence of a structural and functional interaction between P2X_4_ and P2X_7_ receptors (Boumechache et al., 2009). Other P2X receptor subunits form both homomeric or heteromeric receptors such as: P2X_1/2_, P2X_1/4_, P2X_1/5_, P2X_2/3_, P2X_2/5_, P2X_2/6_ and P2X_4/6_ receptors (Jiang et al., 2013). P2X receptors are widely distributed in neuronal and non-neuronal cells, and participate in many physiological and pathophysiological processes (Caseley et al., 2014). Countless *in vitro* and *in vivo* studies have shown changes in these receptors' expression under pain sensation, inflammation and nerve transmission conditions (Jacobson and Muller, 2016). In the CNS, P2X receptors, especially P2X_4_ and P2X_7_ contribute to modulation of neuron-glia communication, inflammation, and apoptosis (Burnstock, 2006b; Surprenant and North, 2009). With the exception of P2X_7_R, other P2XRs subtypes are typically activated at low micromolar (μM) or high nanomolar (nM) concentrations of ATP. Several publications have extensively discussed the distribution, pharmacological properties, physiological and pathophysiological functions of the P2X receptors (Surprenant and North, 2009; Jiang, 2012; Jiang et al., 2013; North and Jarvis, 2013).

### P2X_7_ Receptors

P2X_7_ receptors are members of the P2X family of trimeric ligand-gated cation channel receptors encoded by the P2RX_7_ gene and share the least homology (35–40%) with other P2XRs (North, 2002; Sperlagh et al., 2006). P2X_7_R has the largest monomeric subunit in the P2X family (North, 2002). Each subunit has a short intracellular amino, long carboxyl termini that seems to be essential for most of the receptor activities and two hydrophobic transmembrane domains that are separated by a long glycosylated extracellular ATP-binding domain (Nicke, 2008; Jiang et al., 2013). The C-terminus also plays a role in positioning of the receptor in membrane micro-domains (Murrell-Lagnado, 2017) and/or signaling complexes (Kim et al., 2001; Kopp et al., 2019). Functional P2X_7_ receptor forms a homo-trimeric structure (Jiang et al., 2013), and hold important physiological functions that distinguish it from the other receptor in its family (North, 2002; Sperlagh et al., 2006).

### P2X_7_ Receptor Expression and Activation in the CNS

P2X_7_ receptors are expressed in a number of cell types in the mammalian system including the peripheral and central nervous systems cells (Burnstock, 2008). In the CNS, the highest concentrations of the receptor is expressed on microglia (Weisman et al., 2012; Bhattacharya and Biber, 2016), astrocytes (Ballerini et al., 1996), and oligodendrocytes (Matute et al., 2007) as well as glutamatergic pyramidal neurons of the hippocampus (Metzger et al., 2017). While, P2X_7_R expression on neurons is controversial (Illes et al., 2017), it has been detected on some populations of the spinal cord, cerebellum, hypothalamus, and substantia nigra neurons (Bartlett et al., 2014). Activation of P2X_7_ receptor on microglia subsequently prompts activation of the NLRP3 inflammasome, which induces the release of pro-inflammatory cytokines IL-1β and IL-18, the key mediators of chronic inflammation (Beamer et al., 2016), chronic pain (Bartlett et al., 2014; Beamer et al., 2016; He et al., 2017), neuroinflammation (Monif et al., 2010; Bhattacharya et al., 2013; He et al., 2017), and inflammatory cell death (Leeson et al., 2019). Additionally, activation of P2X_7_R on microglia induces the release of TNFα, production of reactive oxygen species (ROS), and in particular oxygen superoxides, which stimulate the NFκB signaling, release of more pro-inflammatory and pro-apoptotic genes, causing cell-death of surrounding neurons (Parvathenani et al., 2003). Differently from other P2Xs, the P2X_7_ receptor is generally known for its inactivity in normal conditions and activation under toxic effects of high extracellular ATP and therefore, is known as a toxic or death-inducing receptor (Di Virgilio, 2020). P2X_7_R is only activated after injury, infection, in tumor microenvironments, or in conditions that cause increase in extracellular ATP concentration (in the millimolar range of EC_50_ ≥ 100 μM) (Surprenant et al., 1996; Donnelly-Roberts et al., 2009; Bianchi et al., 2014; Fiebich et al., 2014). P2X_7_ receptor is also activated when ectonucleotidases, which degrade ATP and other nucleotides, are downregulated (Di Virgilio et al., 2009; Bartlett et al., 2014). Short exposure of the receptor to ATP results in the channel opening to small cations, including Ca^2+^, Mg^2+^, Na^+^, and K^+^ (Surprenant et al., 1996; Bartlett et al., 2014), while sustained exposure of the receptor to ATP leads to formation of larger pores that allow for the uptake of large organic ions of up to 900 Da (Volonte et al., 2012), and has shown to result in inflammation, cytotoxicity (Di Virgilio et al., 2001; Liang and Schwiebert, 2005) and cell death (Surprenant et al., 1996; Illes et al., 2017). Additionally, activation of P2X_7_R for a longer duration allows for recruitment of pannexin pores (Volonte et al., 2012; Sun et al., 2013; Idzko et al., 2014) allowing the release of even larger amounts of ATP and leading to activation of caspases (Franke et al., 2012), and has been shown to lead to neuro-pathology and cell death (Bartlett et al., 2014). ATP removal from the receptor within 10–15 min of the addition, have resulted in resealing of the plasma membrane and recovery of normal cell functions (Di Virgilio et al., 2018). The major site for neuronal P2X_7_ receptor expression appears to be at presynaptic terminals (Miras-Portugal et al., 2003), resulting in this receptor's participation in the release and regulation of neurotransmitters such as GABA and glutamate (Sperlagh et al., 2002).

### P2X_7_ Receptor Polymorphism

The P2RX genes encode for the human P2X receptors, mainly the human P2X_7_ receptor that is highly polymorphic and contains a large set of single nucleotide polymorphisms (SNPs). Genetic association studies suggest that non-synonymous SNPs (NS-SNPs) in the P2RX genes are important genetic factors in susceptibility of individuals to various diseases (Jiang et al., 2013). The disease-associated NS-SNPs have provided novel insights into disease mechanisms associated with these receptors (Sorge et al., 2012). For instance, ATP-induced influx observed in the T357S polymorphism results in a partial loss of function in human monocytes, lymphocytes, and macrophages, and impaired mycobacterial killing (Shemon et al., 2006; Miller et al., 2011). Similarly, the Q460R polymorphism has been associated with major depressive and bipolar disorders (Barden et al., 2006; Lucae et al., 2006), while ATP-induced ethidium uptake assays in HEK293 cells baring this SNP showed a reduction in pore formation (Stokes et al., 2010), subsequent reduced Ca^2+^ flux, and diminished channel currents associated with impaired cellular signaling. Moreover, in the P2X_7_R knock-in mouse model, which harbors the Q460R polymorphism, if co-expressed with the non-polymorphic variants, results in reductions in sleep quality compared with controls (Aprile-Garcia et al., 2016; Metzger et al., 2017). Similarly, the E496A SNP, has also been associated with cancer metastasis and shown to result in impaired ATP-induced ethidium uptake, Ba^2+^ permeation, and induction of apoptosis in human B-lymphocytes (Gu et al., 2001; Ghiringhelli et al., 2009). Moreover, the human A348T SNP has been shown to induce pore formation and IL-1β secretion (Stokes et al., 2010), while only the H521Q has been described as neutral (Wiley et al., 2011). By contrast, the loss-of-function I568N polymorphism has been reported to prevent cell surface expression and cytosolic receptor trafficking (Wiley et al., 2003). Importantly, the P451L SNP results in loss-of-function, and is found in many common murine strains (i.e., AKR/J, C3H/HeJ, C57BL/6, C57BL/10, CBA/J, DBA/1, DBA/2, FVB/NJ, and NZO/HILtJ), but is not observed in rats, humans, or wild derived mouse strains (i.e., CAST/EiJ, WSB/EiJ and PWK/PhJ) (Yang et al., 2021). This SNP impairs ATP-induced cation fluxes, pore formation, externalization, and apoptosis (Schwarz et al., 2012; Rissiek et al., 2015), and has been associated with reduced pain sensitivity and inflammation in these model systems which harbor the mutation (Sorge et al., 2012).

### P2X_7_ Receptor in Different Species

Significant species differences in receptor pharmacology exists in mouse, rat, and human resulting in altered affinity of these receptors for their native ligand (Donnelly-Roberts et al., 2009). Despite this, P2X_7_ receptor activation does occur, resulting in dye uptake, IL-1β release, and initiation of apoptosis (i.e., phosphatidylserine-flip) present in all isoforms (Kopp et al., 2019). In general human, Rhesus macaque, dog, rat, and mouse P2X_7_ receptors share 77–85% sequence homology (Surprenant et al., 1996; Bartlett et al., 2014), which results in variations in the receptor affinities for ATP (Table 1).

**Table 1:** P2X7 Receptor Antagonist and Radioligands and their application in CNS indications.

**P2X_**7**_ Receptor Antagonists** **P2X_**7**_ Receptor Radioligands**	**K_**i**_ (nM)**	**IC_**50**_ (nM)**	**Application in CNS indications**	**References**
A-438079	7.1 ± 0.08 (h) 6.7 ± 0.1 (r)	6.0 ± 0.02 (h) 5.9 ± 0.2 (r) 5.5 ± 0.2 (m)	Studied in PD, epilepsy, depression, anxiety, and bipolar disorders. Maintained striatal dopamine; but did not prevent the loss of dopaminergic cells in the 6-OHDA model. Reduced noxious and innocuous evoked activity of different classes of spinal neurons in neuropathic rat model. Suppressed seizures and exhibited neuroprotective effects in immature rats. Reduced induced status epileptic seizure. Exhibited antidepressant effects in chronic unpredictable mild stress (CUMS) mice model of depression.	Nelson et al., 2006 Donnelly-Roberts et al., 2009 Bhattacharya et al., 2013 Marcellino et al., 2010 Hracsko et al., 2011 Mesuret et al., 2014 Henshall et al., 2013 Engel et al., 2016 Yue et al., 2017 Bhattacharya et al., 2018 Park and Kim, 2017 Ribeiro et al., 2019
A-839977		20 - 150 (h, r, m)	Produced antinociception in animal models of inflammatory pain. Reduced thermal hyperalgesia in rats. Produced antihyperalgesia in the model of inflammatory pain in mice. Did not induce antihyperalgesic effects in IL-1 knockout mice.	Florjancic et al., 2008 Honore et al., 2009 Friedle et al., 2010
A-740003 [^3^H]A-740003 [^11^C]A-740003		40 (h) 20 (r)	Claimed to be brain-permeable, persisting in brain tissues at least for 1 h after administration. But has already been shown to not enter the brain. Reduced neuropathic pain in rat. Radioligand [^3^H]A-740003 was used in *in vitro* study in post mortem brain sections of MS patients and rat brain sections of a rat model of EAE model of MS.	Honore et al., 2006 Janssen et al., 2014 Beaino et al., 2017 Zarrinmayeh and Territo, 2020
A-804598 [^3^H]A-804598 [^18^F]EFB	8.0 ± 0.04 (h) 8.8 ± 0.06 (r)	7.7 ± 0.13 (h) 6.8 ± 0.17 (r) 7.0 ± 0.06 (m)	Prevented stress-induced depressive-like and anxiety-like behaviors in mice and rats. Induced antidepressant-like effects in FST mice model of depression. Failed to reverse behavioral changes caused by foot shocks in rat. Decreased hepatic inflammation in mice fed a high fat diet and ethanol. Reduced inflammatory markers in hippocampus without altering many neurotransmitters. Converted to [^3^H]A-804598 radioligand to study recombinant rat receptors expressed in 1321N1 cells. PET radioligand [^18^F]EFB,a fluorinated analog of A-804598, showed limited yet quantifiable brain penetration.	Donnelly-Roberts et al., 2009 Bhattacharya et al., 2013 Karasawa and Kawate, 2016 Fabbrizio et al., 2017 Ly et al., 2020 Ruiz-Ruiz et al., 2020 Freire et al., 2019 Ribeiro et al., 2019 Zarrinmayeh and Territo, 2020
GSK1482160 [^11^C]GSK1482160 [^18^F]IUR-1601	2.63 (h)	8.5 (h) 6.5 (r)	Brain penetrable. Entered phase I for treating inflammatory pain in arthritis, but failed to proceed further. PET radioligand [^11^C]GSK1482160 showed high affinity to brain P2X_7_R. PET radioligand [^18^F]IUR-1601 showed similar affinity and selectivity for P2X_7_R as [^11^C]GSK1482160.	Abdi et al., 2010 Ali et al., 2013 Territo et al., 2017 Glaxosmithkline, 2009 Zarrinmayeh and Territo, 2020 Gao et al., 2018
SMW139 [^11^C]SMW139	32 (h)		Entered clinical trial for evaluation of neuroinflammation in MS patients. PET radioligand [^11^C]SMW139 was developed to study *in vivo* marker of neuroinflammation in multiple sclerosis.	Janssen et al., 2018 Hospital, 2019 Hagens et al., 2020 Zarrinmayeh and Territo, 2020
JNJ-42253432	7.9 ± 0.08 (h) 9.1 ± 0.07 (r)	7.7 ± 0.07 (h) 7.8 ± 0.1 (r) 7.1 ± 0.2 (m)	Brain permeable (Brain/Palms = 1). Significantly reduced severe convulsive seizures after one-week treatment.	Lord et al., 2014 Amhaoul et al., 2016
JNJ-47965567	7.9 ± 0.07 (h) 8.7 ± 0.07 (r)	8.3 ± 0.08 (h) 7.2 ± 0.08 (r) 7.5 ± 0.1 (m)	Brain penetrable. Blocked the Bz-ATP induced IL-1β release. Attenuated amphetamine-induced hyperactivity. Efficacious in rat model of neuropathic pain. Reduced temporal lobe epilepsy. Produced long-lasting delay in kindling development. Chronic administration to SOD mice model of ALS modified disease progression in female animals, but had no effect in male animals.	Bhattacharya et al., 2013 Jimenez-Pacheco et al., 2016 Ruiz-Ruiz et al., 2020 Fabbrizio et al., 2017 Ly et al., 2020
JNJ-55308942	8.12 ± 0.08 (h) 8.5 ± 0.04 (r)	7.87 ± 0.2 (h) 7.81 ± 0.2 (r) 7.55 ± 0.5 (m) 7.96 ± 0.1 (mk) 7.72 ± 0.06 (d)	Brain-penetrant (brain/plasma = 1) Engaged brain targets, modulated microglial activation and reduced IL-1β release. Efficacious in models of anhedonia in rodents. Entered phase I clinical trial and is currently in clinical development to study P2X_7_R occupancy in the brain. In clinical trial to assess the safety, tolerability, and pharmacokinetics in healthy participants after administration of single and multiple oral doses.	Ali et al., 2013 Letavic et al., 2017 Bhattacharya, 2018 Chrovian et al., 2018 Letavic et al., 2017 Bhattacharya, 2018 Nv, 2017b Watch, 2021
JNJ-54175446	8.3 ± 0.1 (h) 8.3 ± 0.05 (r)	8.46 ± 0.36 (h) 8.81 ± 0.24 (r)	Brain-penetrant. Entered phase I clinical trial to study safety, tolerability, and pharmacodynamics in participants with major depressive disorder.	Letavic et al., 2017 Kolb et al., 2019 Bhattacharya, 2018 Cctu-Core, 2019
JNJ-64413739 [^18^F]JNJ-64413739	15.9 (h) 2.7 (rat cortex)	1.0 ± 0.2 (h) 2.0 ± 0.6 (r)	Potent, selective and brain permeable. PET radioligand [^18^F]JNJ-64413739 was used to study P2X_7_R in human brain, and can be used for testing target engagement of other brain permeable P2X_7_ antagonists.	Kolb et al., 2019 Koole et al., 2019 Nv, 2017a Zarrinmayeh and Territo, 2020
JNJ-54232334 [^3^H] JNJ-54232334	7.8 ± 0.05 (h) 9.3 ± 0.1 (r)	9.5 ± 0.02 (h) 7.5 ± 0.02 (r)	Higher levels of P2X_7_ *ex vivo* occupancy were measured using [^3^H] JNJ-54232334 due to less non-specific binding. Radioligand [^3^H] JNJ-54232334 showed improved properties over [^3^H] A-804598.	Lord et al., 2015 Rudolph et al., 2015 Zarrinmayeh and Territo, 2020
JNJ-54140515	7.7 (h) 8.9 ± 0.01 (r)	7.7 ± 2.6 (h) 8 ± 2.9 (r)	Readily crossed the blood-brain barrier. Studied brain P2X_7_R occupancy. Has shown a 10-fold increase in brain penetration over JNJ-54232334.	Lord et al., 2015 Bhattacharya and Jones, 2018 Hempel et al., 2013 Rudolph et al., 2015
JNJ-54173717 [^11^C]-JNJ-54173717	1.6 ± 0.1 nM rat cortex	4.2 ± 0.01 (h) 7.6 ± 0.01 (r)	Brain-penetrable. Studied in models of depression, epilepsy and PD. PET radioligand [^11^C]JNJ-54173717 used for studying the brain P2X_7_R functions in both rats and nonhuman primates. [^11^C]JNJ54173717 was also studies in healthy volunteers and PD patients in human.	Ory et al., 2016 Rudolph et al., 2015 Savall et al., 2015 Van Weehaeghe et al., 2019. Zarrinmayeh and Territo, 2020
JNJ-54166060	7 (h) 8 (r)	4 (h) 115 (r) 72 (m)	Brain-penetrable with brain/plasma ratio (~3). Bioavailable P2X_7_R antagonist with moderate clearance. Exhibited dose dependent occupancy in the rat brain with an ED_50_ = 2.3 mg/kg.	Swanson et al., 2016

*P2X_7_R, P2X_7_ receptor; h, human; r, rat; m, mouse; d, dog; mk, monkey; PD, Parkinson's disease; 6-OHDA, 6-hydroxydopamine; CUMS, chronic unpredictable mild stress; PET, positron emission tomography; Bz-ATP, 3′-O-(4-benzoylbenzoyl)-adenosine 5′-triphosphate; SOD, superoxide dismuatase; ALS, Amyotrophic Lateral Sclerosis*.

### P2X_7_ Receptor in Neurodegeneration

**Figure 1:**
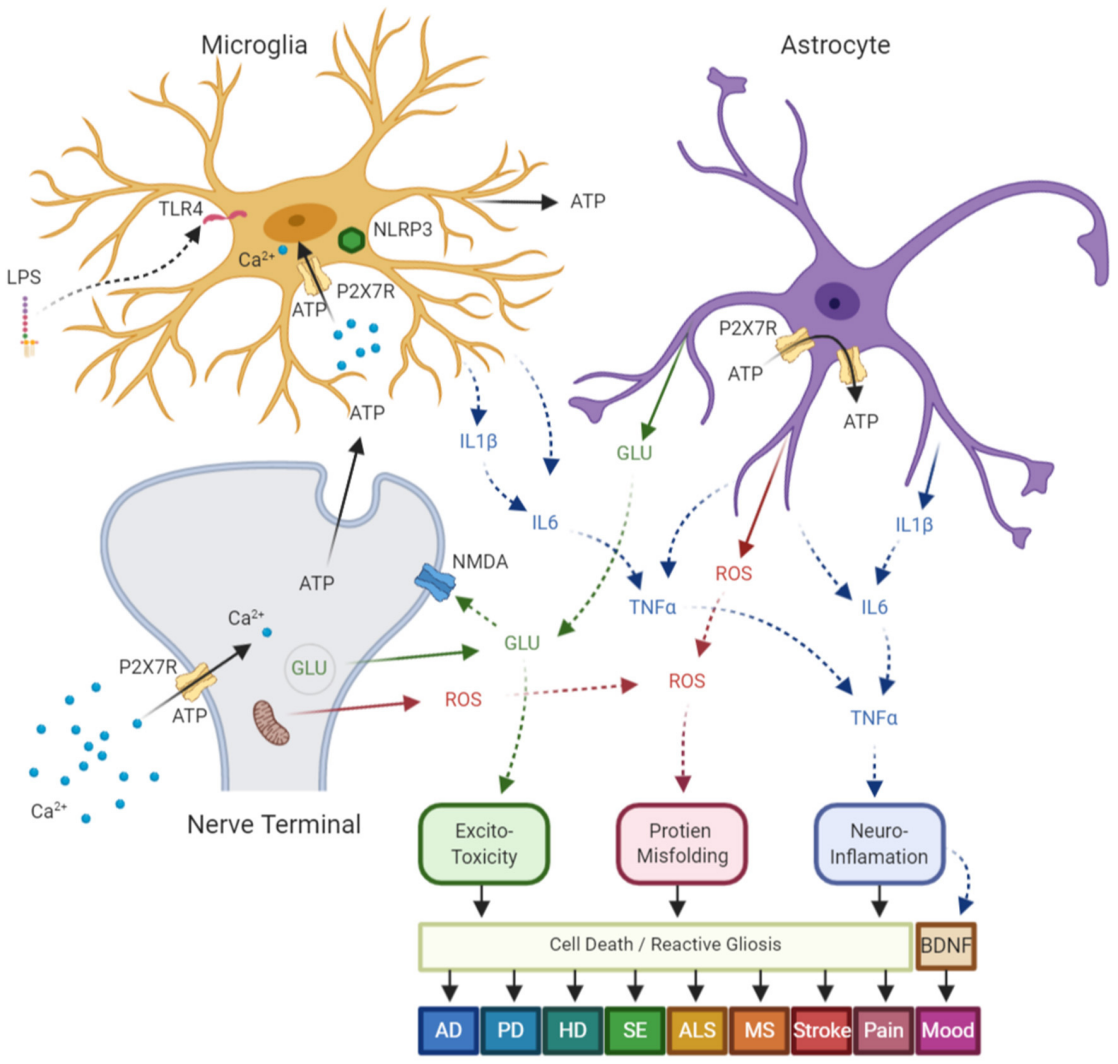
Diagram of common neurologic diseases mediated via P2X_7_ receptors (P2X_7_R)- in central nervous system (CNS). P2X_7_R are expressed on a number of CNS cells, which include: nerve terminals, astrocytes, and microglia. These receptors are upregulated in response to stress signals such as mechanical injury, bacterial or chemical toxins, and hypoxia/ischemia, and lead to a self-amplified release of ATP, which results in further activation of P2X_7_R on neighboring cells. Following ATP dependent Ca^2+^ influx through the receptor ion channel complex, P2X_7_R activation results in: (1) releases glutamate from nerve terminals and astrocytes by both exo and endocytotic mechanisms, which results in excite-toxicity; (2) synthesis and post-translational modification of pro-interleukin-1β (pro-IL-1β) which leads to mature IL-1β and ultimate release via the NLRP3 inflammasome. This activation then leads to other cytokine release and activation leading to neuroinflammation; (3) enhance reactive oxygen and nitrogen species which results in neuronal damage and protein misfolding; which in turn (4) leads to cell death and reactive astrogliosis; and (5) the downregulation of brain-derived neurotrophic factor (BDNF) and alterations in neuronal plasticity. The aforementioned mechanisms have been shown independently, or in concert, to contribute to disease pathology in Alzheimer's disease (AD), Parkinson's disease (PD), Huntington's disease (HD), status epilepticus (SE), amyotrophic lateral sclerosis (ALS), multiple sclerosis (MS), stroke, pain, and mood disorders. ATP, Adenosine Triphosphate; GLU, glutamate; ROS, reactive oxygen species. Figure adapted from Sperlagh and Illes (2014) with modification, and generated using the online software at BioRender.com.

Neurodegenerative diseases (ND) are a cluster of disorders caused by either hereditary or sporadic conditions and characterized by progressive dysfunction of the nervous system that leads to inflammation, gliosis and degeneration of neurons in the brain and/or the spinal cord (Kanellopoulos and Delarasse, 2019). In addition to aging which is the main contributor to the neurodegenerative diseases, abnormal protein aggregation in the brain cells represents a common hallmark in these pathologies as seen in both Alzheimer's disease (AD) and Parkinson's disease (PD), the two most common neurodegenerative disorders. Neuroinflammation, the harmful shared characteristic of neuronal degeneration is the consequence of P2X_7_R activation on microglia (Sperlagh et al., 2006; Burnstock et al., 2011; Sperlagh and Illes, 2014) (Figure 1). ATP-dependent P2X_7_Rs activation induce necrosis by encouraging membrane pores opening that subsequently promote loss of intracellular proteins and promote activation of the caspase pathway, bringing apoptosis to glial cells. P2X_7_Rs driven microglial activation has been implicated in neuroinflammation and neurodegeneration (Monif et al., 2010; Illes et al., 2017). Therefore, P2X_7_ receptor modulation has proven to be a promising option for treatment of neurodegenerative diseases and antagonists of this receptor have shown to slow deposition of the amyloid plaque and progression of AD disease in animal model systems (Diaz-Hernandez et al., 2012; Rodrigues et al., 2015).

### P2X_7_ Receptor in Alzheimer's Disease

The appearance of plaques that consist of extracellular β-amyloid (Aβ) peptide surrounded by reactive microglial is a major hallmark of AD (Sanz et al., 2009). Aβ peptide, produced via cleavage of the amyloid precursor protein (APP) by β- and γ-secretases has shown to trigger increases in Ca^2+^, ATP and IL-1β, induce plasma membrane permeabilization and consecutively damage neurons (Delarasse et al., 2011; Martin et al., 2019) (Figure 1). P2X_7_ receptor has shown to be upregulated on activated microglia in the hippocampus of Tg2576 transgenic mice models of AD, and in rat's brain following intra-hippocampal Aβ injection (Parvathenani et al., 2003; Mclarnon et al., 2006). Additionally, P2X_7_ receptor deficient microglia was not activated with Aβ (Sanz et al., 2009); P2X_7_ receptor silencing enhanced microglia phagocytosis and clearance of Aβ (Ni et al., 2013); and receptor deficiency reduced Aβ plaque, improve synaptic plasticity and resulted in reduction of cognitive deficits in AD mice model (Chen et al., 2014). The P2X_7_R antagonist BBG has shown to decrease Aβ plaque buildup in hippocampal neurons and improve cognition in J20 mice model of AD (Chen et al., 2014). This effect has been due to the boost in α-secretase activity by BBG (a P2X_7_R antagonist) via reduction of GSK3β activity (Diaz-Hernandez et al., 2012), an APP phosphorylating enzyme (Mclarnon et al., 2006; Ryu and Mclarnon, 2008). However, in AD mice, the absence of P2X_7_R did not influence IL-1β release or non-amyloidogenic fragment sAPPα level (Martin et al., 2019). Furthermore, *in vitro* studies has indicated that stimulation P2X_7_R for a short time (~30 minutes) increased α-secretase activity (Delarasse et al., 2011). Therefore, the involvement of P2X_7_R in AD is still somewhat unclear and requires more investigation.

### P2X_7_ Receptor in Parkinson's Disease

P2X_7_-induced microglia activation has been detected in PD pathology (Carmo et al., 2014). α-Synuclein binding to, and activating of the P2X_7_ receptor on microglia (Jiang et al., 2015; Wilkaniec et al., 2017) have been detected in the brains of patients with PD (Durrenberger et al., 2012; Jiang et al., 2015). P2X_7_ receptor has also been shown to participate in the nigrostriatal degeneration in rat model of PD. Motor and memory deficit induced by 6-hydroxydopamine (6-OHDA) animal model of PD was ameliorated by administration of selective P2X_7_ receptor antagonists (Marcellino et al., 2010; Carmo et al., 2014; Ferrazoli et al., 2017; Kumar et al., 2017), suggesting P2X_7_ receptor play a pro-inflammatory role in microglia activation in PD (Kumar et al., 2017). Additionally, P2X_7_ receptor antagonist BBG significantly prevented, and in some cases reversed loss of dopaminergic neurons in the 6-OHDA model (Carmo et al., 2014; Ferrazoli et al., 2017). However, A-438079, another P2X_7_R antagonist maintained striatal dopamine; but, did not prevent the loss of dopaminergic cells in the 6-OHDA model (Marcellino et al., 2010). Furthermore, inhibition and/or genetic deletion of P2X_7_R did not induce neuro-protection in the MPTP mouse model of Parkinson's disease (Hracsko et al., 2011). More research is needed to further clarify the relationship between P2X_7_R activation and PD.

### P2X_7_ Receptor in Multiple Sclerosis

Multiple sclerosis (MS) is an inherited degenerative disease, which results in focal inflammatory lesions in both white and gray matter (Hagens et al., 2020). MS is caused by immune cell infiltration, loss of oligodendrocytes, axonal damage, demyelination, and neuronal death. Increased level of P2X_7_ receptor expression has been detected on microglia (Yiangou et al., 2006), astrocytes (Narcisse et al., 2005), and oligodendrocytes (Matute et al., 2007) of the post-mortem multiple sclerosis patients (Narcisse et al., 2005; Grygorowicz et al., 2010; Burnstock, 2015; Sadovnick et al., 2017). In the acute phase of the disease, elevated expression of P2X_7_R have been observed in neurons, astrocytes and oligodendrocytes, causing the release the pro-inflammatory cytokines that has shown to contribute to the progressive inflammation, degeneration and cells death in Experimental Autoimmune Encephalomyelitis (EAE) model of MS (Grygorowicz et al., 2016) (Figure 1). Remarkably, P2X_7_R expression is down-regulated on peripheral monocytes of MS patients during the acute phase of the disease (Amadio et al., 2017). This behavior of the receptor has created a gap in understanding of the receptor function in MS and the treatment of the disease. Several cohort studies have acknowledged an association of loss-of-function, minor allele frequency P2X_7_ receptor SNP, rs28360457, coding for R307G with protection against MS and a gain-of-function haplotype rs208294, coding for H155T, which increases risk of MS (Oyanguren-Desez et al., 2011; Gu et al., 2015). Additionally, in a case-control study, elevated frequency of a gain-of-function SNP, rs17525809 coding for A76V in MS patients was observed (Oyanguren-Desez et al., 2011), suggesting that P2X_7_R variants may play a significant role in the pathogenesis of MS disease. Moreover, studies have reported that P2X_7_ receptor antagonist decreased astrogliosis, abridged demyelination, and improved neurological symptoms in an EAE rat model of MS (Grygorowicz et al., 2016). Some of these antagonists, including BBG, have shown to improve motor deficits in EAE by decreasing demyelination that consequently improves axonal conduction (Bartlett et al., 2017).

### P2X_7_ Receptor in Huntington's Disease

Huntington's disease (HD) is an inherited neurodegenerative disorder caused by progressive degeneration of nerve cells in the cortex and striatum of the brain (Oliveira-Giacomelli et al., 2018). Patients with HD experience progressive cognitive decline in addition to motor and psychological dysfunction. Brains of Tet/HD94 and R6/1 mice, which are genetic models of HD, have shown increased mRNA and protein levels of P2X_7_ receptor (Diaz-Hernandez et al., 2009), and BBG has shown to prevent loss of body weight and motor coordination deterioration in the R6/1 mouse model of HD (Diaz-Hernandez et al., 2009). Importantly, recent work by Olla et al. (2020) has shown that P2X_7_R has been found upregulated, and in some case showed altered splicing, in the brain of HD subjects.

### P2X_7_ Receptor in Amyotrophic Lateral Sclerosis

Amyotrophic lateral sclerosis (ALS) is an adult-onset neurodegenerative disease characterized by decline and loss of motor neurons (i.e., both upper and lower) (Oliveira-Giacomelli et al., 2018). Inflammation and autophagy play critical roles in the pathogenesis of ALS, while several studies have implicated the role of P2X_7_Rs in pathogenesis (Volonte et al., 2016). Increased expression of P2X_7_Rs has been detected in microglia (D'ambrosi et al., 2009; Rudnick et al., 2017) or astrocytes (Gandelman et al., 2010; Apolloni et al., 2014) isolated from superoxide dismutase 1 (SOD1^G93A^) mouse model of ALS, and application of the P2X_7_R antagonist BBG improved spinal cord pathology and ameliorated the disease in these mice (Apolloni et al., 2014; Bartlett et al., 2017). Additionally, more potent and selective P2X_7_R antagonists such as A804598 and JNJ-47965567 have provided some beneficial effects in ALS mouse models (Fabbrizio et al., 2017; Ly et al., 2020; Ruiz-Ruiz et al., 2020). Furthermore, ATP-induced activation of P2X_7_R has shown to activate kinase ERK1/2 and NOX2 in microglia of SOD1^G93A^ mice (Apolloni et al., 2013). Nevertheless, P2X_7_R down-regulation has also been detected in peripheral circulating monocytes of ALS patients (Liu et al., 2016), which contrasts the up-regulation of the receptor in spinal cord and nervous tissues of post-mortem ALS patients post-mortem (Yiangou et al., 2006). Thus far, studies of the P2X_7_R function in ALS has not been conclusive and it seems that this receptor plays a dual role in ALS (Volonte et al., 2020).

### P2X_7_ Receptor in Epilepsy

Epilepsy can occur from an insult to the brain (stroke and head trauma such as in TBI), repeated episodes of status epilepticus or genetic malfunction (Rees, 2010; Pitkanen and Lukasiuk, 2011). In adults, the most common form of acquired epilepsy is temporal lobe epilepsy (TLE) which is characterized by a pattern of reactive gliosis and selective neuronal loss, a phenomenon that is also called hippocampal sclerosis (Chang and Lowenstein, 2003). P2X_7_ receptor activation and upregulation has been associated with TLE and inhibition of receptor activity with antagonist JNJ-47965567 has shown to reduce TLE both during and past the time of drug presence (Jimenez-Pacheco et al., 2016). Hippocampal sections of these treated mice displayed reduction of activated microglia and astrocytes, suggesting anticonvulsant and anti-epilepsy property of P2X_7_R antagonists (Jimenez-Pacheco et al., 2016). BBG treatment of rats with spontaneous recurrent seizures helped treatment by reducing the P2X_7_ receptor expression (Fischer et al., 2016; Song et al., 2019). Additionally, significant reduction in severe convulsive seizures was observed after treatment with P2X_7_R antagonist JNJ-42253432 for a week (Amhaoul et al., 2016). Another P2X_7_R antagonist A-438079 suppressed seizures and exhibited neuroprotective effects in immature rats (Mesuret et al., 2014). P2X_7_R antagonists BBG and A-438079 have also been shown to reduce status epileptic seizure caused by unilateral injection of kainic acid into the mice amygdala in rodent model of temporal lobe epilepsy (Henshall et al., 2013; Engel et al., 2016). Additionally, several lines of evidence confirms increase of P2X_7_R in hippocampus of patients with pharmaco-resistant temporal lobe epilepsy and in hippocampal subfields of mice that experienced status epilepticus (Jimenez-Pacheco et al., 2016). These studies support the role of P2X_7_ receptor antagonists in treating epilepsy, including drug-resistant epilepsy (Beamer et al., 2016; Cieslak et al., 2017; Rodriguez-Alvarez et al., 2017). Furthermore, P2X_7_R have been recognized as targets for treatment of hypoxic/ischemic encephalopathy (Beamer et al., 2017). Recent anticonvulsant studies using P2X_7_R antagonists, which include BBG, AFC-5128, JNJ-47965567, and tanshinone IIA sulfonate (traditional Chinese herbal medicine, TIIAS) in animal models have illustrated the potential of these agents to modulate seizures. Remarkably in the pentylenetetrazol-kindling (PTZ-kindling) and maximal electroshock seizure (MES) models threshold test, none of the compounds showed anticonvulsant effects when given by itself; however, when given in combination with carbamazepine, AFC-5128 and JNJ-47965567 increased the threshold in the MES test (Fischer et al., 2016). Similarly, in the PTZ-kindling rat model anti-epileptogenic activities for BBG and TIIAS were observed, whereas the P2X_7_R inhibitors AFC-5128 and JNJ-47965567 showed long-lasting delay in kindling development, while results in fully kindled animal showed reductions in seizure stage (Fischer et al., 2016). In the case of epilepsy, P2X_7_ receptors have three distinct functions depending on the situations of the extracellular environment. P2X_7_R initiates cell death in the presence of elevated extracellular ATP, while mediates calcium signal transduction in response to ATP that regulates proliferation and differentiation. Finally, P2X_7_R activation promotes phagocytosis in the absence of extracellular ATP (Zheng et al., 2017).

### P2X_7_ Receptor in Ischemia, Stroke and Trauma

Ischemic stroke, one of the major type of strokes, results from oxygen and glucose deprivation that cause cell death (Hempel et al., 2013; Yoshida et al., 2015). Neuroinflammation and over expression of the P2X_7_ receptor has been detected in this ischemic stroke (Figure 1) and the neuroprotective effects of P2X_7_R suppression has been proven to be successful in this type of stroke (Melani et al., 2006; Eyo et al., 2013). The ATP degrading enzyme apyrase and P2X_7_ receptor antagonists have shown to relieve the damage initiated by ischemia and improve action potential recovery (Domercq et al., 2010). In hemorrhagic stroke, where a sudden rupture of cerebral blood vessels and cell death promote quick release and accumulation of large quantity of ATP, P2X_7_R inhibition has been promising in prevention of acute neuroinflammation and cell death (Chen et al., 2013a,b). Striatal P2X_7_R has also shown to intensify neuroinflammation and brain damage in intracerebral hemorrhage (ICH) possibly via activation of NLRP3 inflammasome and release of IL-1β/IL-18. The P2X_7_R antagonist BBG treatment following ICH has shown to downregulate the release of these proinflammatory cytokines (Feng et al., 2015). Inhibition of P2X_7_Rs improved global cerebral ischemia/reperfusion injury. This positive effect is evidenced by increase in survival rate, reduction of neuronal death in the hippocampal CA1 region, and improvement in learning memory (Chu et al., 2012; Yu et al., 2013). P2X_7_Rs are also involved in cerebral neurological damage and edema after traumatic brain injury. Application of P2X_7_R antagonist BBG has resulted in decreased expression of Glial fibrillary acidic protein (GFAP) and reduction of aquaporin-4, which is an astrocytic water channel that promotes cellular edema (Kimbler et al., 2012; Leeson et al., 2019).

### P2X_7_ Receptor in Depression

P2X_7_ receptor activation by ATP followed by NLRP3 induced IL-1β release that results in neuroinflammation are major contributors of neuropsychiatric disorders, especially depression (Adinolfi et al., 2018; Bhattacharya and Jones, 2018; Franklin et al., 2018; Li and Barres, 2018; Liu et al., 2018). An anti-inflammatory Chinese medicine, Chrysophanol has shown an anti-depressant effects by mediation of the P2X_7_R/NFκB signaling pathway (Zhang et al., 2016) supporting the role of P2X_7_ receptor participation in depression. Additionally, the P2X_7_R knock out mice did not show signs of depression in forced swim and tail suspension tests (Basso et al., 2009; Leeson et al., 2019), consistent with this hypothesis. Some brain penetrable P2X_7_R antagonists such as BBG and A-438079 have exhibited antidepressant effects in chronic unpredictable mild stress (CUMS) mice model of depression by inhibiting the activation of P2X_7_/NLRP3/IL-1β pathway (Yue et al., 2017; Bhattacharya, 2018). Furthermore, stress is known to prompt production of excessive glutamate that is proven to stimulate large ATP release from astrocytes, activating P2X_7_R and subsequently increasing IL-1β level in the brain (Faloia et al., 2012).

## P2X_7_ Receptor Ligands

### Agonists

Two major agonist of the P2X_7_ receptor are ATP and BzATP, which is 10–30 fold more potent than ATP (Surprenant et al., 1996; Beigi et al., 2003), but also activates P2X_1_ and P2X_3_ receptors (De Marchi et al., 2016). Activation of P2X_7_R by ATP induces neuroinflammation that results in pathogenesis of many diseases of CNS, suggesting significant potential for P2X_7_ receptor antagonists to combat the diseases of neuroinflammatory origin (Mehta et al., 2014). Therefore, there has been extensive effort to develop several potent and selective P2X_7_R antagonists.

### Antagonists

There are two non-selective ATP derivative antagonists TNP-ATP and periodate-oxidized ATP (oATP) with high μmol potencies (Beigi et al., 2003; Di Virgilio, 2003; De Marchi et al., 2016). The major group of P2X_7_R antagonists are the non-ATP based compounds. Depending on their interaction with the receptors, some are orthostatic antagonists that bind competitively to the ATP binding pocket, while majority are allosteric antagonists that bind to other locations than the ATP-binding site, and reduce ATP binding affinity to the receptor (De Marchi et al., 2016).

### First Generation Antagonists

The first generation of non-ATP antagonists were mostly designed for *in vitro* study of the receptor and included compounds such as: Reactive Blue 2 (Bartlett et al., 2014), Suramin (Leff et al., 1990), Brilliant Blue G (BBG) (Jiang et al., 2000), the irreversible PPADS (Jiang et al., 2000), and KN-62 (Gever et al., 2006; Bartlett et al., 2014). Among this list, KN-62 (Bartlett et al., 2014) and the non-specific BBG exhibited brain permeability property (Wang et al., 2004, 2017; Donnelly-Roberts et al., 2009; Peng et al., 2009; Jo and Bean, 2011; Carmo et al., 2014). BBG is currently the most widely used P2X_7_R antagonist in research (Peng et al., 2009). BBG has also shown to block pannexin-1 (Bin Dayel et al., 2019). Additional primary antagonists include Chelerythrine and other benzophenanthridine alkaloids (Shemon et al., 2004), CAY10593 which is a synthetic phospholipase D blocker (Pupovac et al., 2013), Ca^2+^ and Mg^2+^ cations (Jiang, 2009). Some of the aforementioned antagonist exhibited poor stability and less desirable pharmacokinetics properties, preventing their use for *in vivo* studies of the receptor (Jiang, 2012).

### Second Generation Antagonists

The second generation of the P2X_7_R antagonists were developed aiming for higher potency, selectivity, *in vivo* stability and possible equal potency at different species. Among this list are the tetrazole-based compounds A-438079 and A-839977 (Table 1). Antagonist A-438079 [K_i_ = 7.1 ± 0.08 (h) and K_i_ = 6.7 ± 0.1 (r); IC_50_ = 6.0 ± 0.02 (h), IC_50_ = 5.9 ± 0.2 nM (r), and IC_50_ = 5.5 ± 0.2 nM (m)] (Nelson et al., 2006; Donnelly-Roberts et al., 2009; Bhattacharya et al., 2013) has been studied in PD (Marcellino et al., 2010) and while maintained striatal dopamine, it did not prevent the loss of dopaminergic cells in the 6-OHDA model of PD (Marcellino et al., 2010). It suppressed seizures and exhibited neuroprotective effects in immature rats (Mesuret et al., 2014). A-438079, also exhibited antidepressant effects in chronic unpredictable mild stress (CUMS) mice model of depression (Yue et al., 2017; Bhattacharya, 2018). Another tetrazole based antagonist A-839977 [IC_50_ = 20–150 nM (h r, m)] reduced thermal hyperalgesia in rats and produced antihyperalgesia in the CFA model of inflammatory pain in mice (Florjancic et al., 2008; Honore et al., 2009; Friedle et al., 2010). However, A-839977 did not induce any antihyperalgesic effects in IL-1 knockout mice (Honore et al., 2009).

Another class of antagonists in the second generation group are the cyanoguanidine based compounds A-740003 and A-804598 (Table 1). Cyanoguanidine A-740003 [IC_50_ = 40 nM (h) and IC_50_ = 20 nM at (r)] dose-dependently reduces neuropathic pain in rat (Honore et al., 2006). This antagonist was converted to [^11^C]A-740003 PET radioligand and while has been shown not to enter the brain (Janssen et al., 2014), its tritiated analog [^3^H]A-740003 was used in an *in vitro* study in post mortem brain sections of MS patients and rat brain sections of a rat model of EAE model of MS (Beaino et al., 2017). Antagonist A-804598 [K_i_ = 8.0 ± 0.04 nM (h), K_i_ = 8.8 ± 0.06 nM (r); IC_50_ = 7.7 ± 0.13 nM (h), IC_50_ = 6.8 ± 0.17 nM (r), IC_50_ = 7.0 ± 0.06 nM (m)] (Donnelly-Roberts et al., 2009; Bhattacharya et al., 2013; Karasawa and Kawate, 2016) was used to study the functional role of P2X_7_R in inflammatory response in the liver and brain of the C57BL/6J mice fed a high fat diet and those with chronic ethanol consumption. It showed reduction of inflammatory markers in hippocampus without altering many neurotransmitters and decrease in hepatic inflammation but not steatosis (Freire et al., 2019). A-804598 has also shown to induce antidepressant-like effects in the FST mice model of depression (Ribeiro et al., 2019), but failed to reverse behavioral changes caused by foot shocks in rat (Catanzaro et al., 2014). Tritiated A-804598 ([^3^H]A-804598) was also prepared and utilized as a P2X_7_R radioligand to study recombinant rat receptors expressed in 1321N1 cells (Donnelly-Roberts et al., 2009). Fluorinated analog of A-804598 was also converted to F-18 PET radioligand [^18^F]EFB that showed limited yet quantifiable brain penetration (Zarrinmayeh and Territo, 2020).

Other second-generation antagonists included: AZD9056 (Keystone et al., 2012); AZ-11645373 (Stokes et al., 2006; Syed and Kennedy, 2012; Mehta et al., 2014); AZ-10606120 (Michel et al., 2007); GW791343 (Felix et al., 2012); GSK314181A (Broom et al., 2008); GSK1482160 (Ali et al., 2013); CE-224,535 (Stock et al., 2012); AFC-5128 (Fischer et al., 2016); SMW139 (Hansen et al., 2018); and EVT-401 (Zhu et al., 2017). Among the list of antagonists, AZD9056 (Keystone et al., 2012) and CE-224,535 (Stock et al., 2012) entered clinical trials in patients with rheumatoid arthritis and while passed acceptable safety and tolerability hurdles, they failed in phase II efficacy (Stokes et al., 2006; Keystone et al., 2012). EVT-401 also entered phase I clinical trial in patients with rheumatoid arthritis, but did not advance further (Zhu et al., 2017; Evotec, 2020). GSK1482160 entered phase I clinical trials for treating inflammatory pain in arthritis, but failed to proceed beyond phase I (Glaxosmithkline, 2009). GSK1482160 was also converted to C-11 PET radioligand [^11^C]GSK1482160 and showed high affinity (K_d_ = 1.15 ± 0.12 nM) in targeting P2X_7_R (Territo et al., 2017). Recent work from our lab has also developed an F-18 PET radioligand [^18^F]IUR-1601 that shows similar affinity and selectivity [K_i_ = 4.31 nM (h), IC_50_ = 7.86 nM (h)] for P2X_7_R as [^11^C]GSK1482160 (Gao et al., 2018). Another benzamide, compound, SMW139 has also been converted to PET radioligand [^11^C]SMW139 to study the P2X_7_R receptor expression on pro-inflammatory microglia (Janssen et al., 2018). [^11^C]SMW139 proceeded to first in-man study to evaluate its potential in identifying *in vivo* neuroinflammation in MS patients (Hospital, 2019; Hagens et al., 2020).

### New Generation Antagonists

The newer generation of the P2X_7_R antagonists have specifically been designed to penetrate CNS and enable evaluation of the P2X_7_R functions in the CNS disorders including neuroinflammation (Bhattacharya, 2018). This collection of highly potent and selective antagonists has been clustered into four groups based on their chemical scaffolds. They are presented in Table 1 and are briefly mentioned herein:

#### Group 1

The phenylpiperazine based compounds JNJ-42253432 and JNJ-47965567. JNJ-42253432 is a potent P2X_7_R antagonist [K_i_ = 7.9 nM ± 0.08 (h) and K_i_ = 9.1 ± 0.07 nM (r); IC_50_ = 7.7 ± 0.07 nM (h), IC_50_ = 7.8 ± 0.1 nM (r) and IC_50_ = 7.1 ± 0.2 nM (m)] that penetrates into the CNS (Brain/Plasma = 1) (Lord et al., 2014). With its excellent pharmacokinetic and pharmacodynamic properties, JNJ-42253432 has shown to block the Bz-ATP-induced release of IL-1β in a concentration-dependent manner (Lord et al., 2014). Significant reduction in severe convulsive seizures was also detected after one-week treatment with JNJ-42253432 (Amhaoul et al., 2016). Another centrally permeable phenylpiperazine based antagonist is JNJ-47965567 that has shown high potency [K_i_ = 7.9 ± 0.07 nM (h) and K_i_ = 8.7 ± 0.07 nM (r); IC_50_ = 8.3 ± 0.08 nM (h), IC_50_ = 7.2 ± 0.08 nM (r), and IC_50_ =7.5 ± 0.1 nM (m)]. It exhibited target engagement in rat brain (EC_50_ = 78 ± 19 ng/ml in P2X_7_R autoradiography) and functionally blocked the Bz-ATP induced IL-1β release (Bhattacharya et al., 2013). JNJ-47965567 reduced amphetamine-induced hyperactivity and showed substantial efficacy in neuropathic rat model of pain (Bhattacharya et al., 2013). JNJ-47965567 significantly reduced temporal lobe epilepsy characterized by a pattern of selective neuronal loss and reactive gliosis (Jimenez-Pacheco et al., 2016). Chronic administration of JNJ-47965567 (4X/week) to SOD mice model of ALS modified disease progression in female animals, but had no effect in male animals, suggesting partial effect of P2X_7_R in progression of ALS (Ruiz-Ruiz et al., 2020).

#### Group 2

The 1,2,3-triazolo based antagonists include JNJ-55308942, JNJ-54175446, and JNJ-64413739. JNJ-55308942 is a P2X_7_R antagonist with high potency [K_i_ = 8.12 ± 0.08 nM (h) and 8.5 ± 0.04 nM (r)] (Ali et al., 2013; Letavic et al., 2017; Bhattacharya et al., 2018; Chrovian et al., 2018). JNJ-55308942 is a brain-penetrant antagonist (brain/plasma = 1) that has shown a prominent pharmacology at recombinant human, rat, mouse, macaque, and dog P2X_7_R [IC_50_ = 7.87 ± 0.2 nM (h), IC_50_ = 7.81 ± 0.2 nM (r), IC_50_ = 7.55 ± 0.5 nM (m), IC_50_ = 7.96 ± 0.1 nM (mm), and IC_50_ = 7.72 ± 0.06 (d)] (Letavic et al., 2017; Nv, 2017b; Bhattacharya et al., 2018). JNJ-55308942 has shown to engage brain targets, modulates microglial activation, reduce IL-1β release and has been efficacious in models of anhedonia in rodents (Letavic et al., 2017; Bhattacharya et al., 2018; Chrovian et al., 2018). JNJ-55308942 entered phase I of clinical trial in 2017 to assess the safety, tolerability, and pharmacokinetics in healthy participants after administration of single and multiple oral doses (Nv, 2017a; Watch, 2021). Another 1,2,3-triazolo based P2X_7_R antagonist is the highly potent and brain penetrant JNJ-54175446 [K_i_ = 8.3 ± 0.1 nM (h) and K_i_ = 8.3 ± 0.05 nM (r), IC_50_ = 8.46 ± 0.36 (h), and IC_50_ = 8.81 ± 0.24 (r)] with dose-dependent occupancy (ED_50_ = 0.46 mg/kg, corresponding to plasma EC_50_ = 105 ng/ml) (Kolb et al., 2019). JNJ-54175446 has also entered phase I of clinical trial to study its antidepressant activity (Bhattacharya, 2018; Cctu-Core, 2019). Third 1,2,3-triazole based antagonist is the selective and potent JNJ-64413739 [K_i_ =15.9 nM (h) and K_i_ = 2.7 nM at rat cortex; IC_50_ = 1.0 ± 0.2 nM (h) and IC_50_ = 2.0 ± 0.6 nM (r)] (Kolb et al., 2019; Koole et al., 2019). The F-18 PET radioligand of JNJ-64413739, [^18^F]JNJ-64413739 was developed to study brain P2X_7_ function (Zarrinmayeh and Territo, 2020). Micro-dosing in mice showed a 38% lower compound binding in P2X_7_R knock out compared to wild type mice receptor and the uptake of [^18^F]JNJ-64413739 was reduced by JNJ-54175446 in a dose-related manner in a monkey PET study (Nv, 2017a; Kolb et al., 2019; Koole et al., 2019). Study of [^18^F]JNJ-64413739 in healthy human volunteer also showed the tracer to be an appropriate PET ligand for quantification of P2X_7_R expression in the human brain (Table 1) (Nv, 2017a; Kolb et al., 2019; Koole et al., 2019).

#### Group 3

The 1,2,4-triazolo based P2X_7_R antagonists are three close analogs JNJ-54232334, JNJ-54140515, and JNJ-54173717. The high affinity JNJ-54232334 [K_i_ = 7.8 ± 0.05 nM (h) and K_i_ = 9.3 ± 0.1 nM (r); IC_50_ = 9.5 ± 0.02 nM (h), and IC_50_ = 7.5 ± 0.02 nM (r)] was tritiated to produce [^3^H]JNJ-54232334 that reached saturable binding, and equilibrium dissociation rate constant (*K*_d_) of 4.9 ± 1.3 nM (Lord et al., 2015; Rudolph et al., 2015). The specific binding of [^3^H]JNJ-54232334 in rat brain sections was enhanced compared to that of the [^3^H] A-804598 as a result of low non-specific binding (Lord et al., 2015). JNJ-54140515, an analog of JNJ-54232334 with comparable *in vitro* pharmacology [K_i_ = 7.7 nM (h) and K_i_ = 8.9 ± 0.01 nM (r); IC_50_ = 7.7 ± 2.6 nM (h) and IC_50_ = 8.0 ± 2.9 nM (r)], readily crossed the blood-brain barrier and facilitated the high level of brain P2X_7_R occupancy (Lord et al., 2015; Bhattacharya and Jones, 2018). While similar in potency, JNJ-54140515 has shown a 10-fold increase in brain penetration over JNJ-54232334 (Hempel et al., 2013; Lord et al., 2015; Rudolph et al., 2015). JNJ-54173717 is another high affinity P2X_7_R antagonists at human and rat receptor [K_i_ = 1.6 ± 0.1 nM in rat cortex; IC_50 =_ 4.2 ± 0.01 nM (h) and IC_50_ = 7.6 ± 0.01 nM (r)]. JNJ-54173717 has good drug-like properties and high P2X_7_ receptor occupancy in rat subsequent oral administration (Ory et al., 2016). JNJ-54173717 has been studied in models of depression, epilepsy and PD (Rudolph et al., 2015; Van Weehaeghe et al., 2019). JNJ-54173717 was also converted to PET radioligand [^11^C]JNJ-54173717 for studying the brain P2X_7_R functions in both rats and nonhuman primates (Ory et al., 2016). This tracer crossed the blood-brain barrier, and was cleared from plasma via hepatobiliary pathways in rat bio-distribution study (Rudolph et al., 2015; Savall et al., 2015; Ory et al., 2016). [^11^C]JNJ54173717 was studies in healthy volunteers and PD patients in human and showed selectivity for P2X_7_R (Van Weehaeghe et al., 2019).

#### Group 4

The imidazolopyridin JNJ-54166060 is another P2X_7_R antagonist with high potency [K_i_ = 7 nM (h) and K_i_ = 8 nM (r); IC_50_ = 4 nM (h), IC_50_ = 115 nM (r), and IC_50_ = 72 nM (m)] that has shown great oral bioavailability and low-moderate clearance in preclinical animal models. It has brain penetrable property, exhibiting a significant brain/plasma ratio (~3) with ED_50_ = 2.3 mg/kg when dosed orally (Swanson et al., 2016).

### Controversies, Research Gaps, and Potential Developments

While numerous research studies have been conducted to unravel the function of P2X_7_R in neurodegenerative disorders, there are still unanswered questions that could add clarity to our understanding of the role of P2X_7_R in CNS disease progression. For example, in patients with MS, P2X_7_R expression is down-regulated on peripheral monocytes during the acute phase of the disease (Amadio et al., 2017), while it appears to be up regulated on myeloid derived cells in the CNS. Similarly, P2X_7_R down-regulation has also been detected in peripheral circulating monocytes of ALS patients (Liu et al., 2016), while this contrasts the up-regulation of the receptor in spinal cord and nervous tissues of post-mortem ALS patients (Yiangou et al., 2006). The fact that P2X_7_R shows opposite expression patterns in peripheral vs. central compartments, and in some cases shows a temporal expression pattern which is also tissue specific, suggest that additional work is needed to better understand the interdependency of these compartments on disease progression. Another aspect of P2X_7_R expression is related to the genetic context in which the receptor is being expressed. As noted above, many of the mouse models of AD, PD, HD, ALS, MS, TBI, stroke, and depression have been conducted in mice which bares the P451L SNP, as such it is unclear if these mice show the full complement of signaling associated with the P2X_7_R system, since the polymorphism results in reduction in sensitivity to ATP by several orders of magnitude. Based on work from our laboratory (Territo et al., 2017), we showed that activation of P2X_7_R in C57BL6/J mice required 5–10 fold higher levels of lipopolysaccharide than have been reported for mice which do not carry this polymorphism. Provided this, we believe that both face and construct validity are required in model systems to ensure that they faithfully replicate the type of signaling observed in human cell lines and clinical studies (see Table 1). Therefore, to better understand the role P2X_7_R in neurodegenerative diseases, we believe it is imperative that one asks the right question, at the right time, and using the right model systems to maximizes our understanding so that these results can be used to help inform and guide clinical trials.

## Concluding Remarks

Purinergic receptors (i.e., purinoceptors) are plasma membrane proteins that play key physiological roles in mammalian central nervous system (CNS), and regulate neurotransmission, neuromodulation, and intra and inter-glial network communication. Purinoceptors are found in a variety of cells in the CNS that include microglia, astrocytes and oligodendrocytes. Astrocytes express many types of purinergic receptors, and release adenosine triphosphate (ATP) as an intercellular signaling molecule allowing communication of these cells with microglia, neurons, oligodendrocytes and the vascular walls of capillaries. In the CNS, purinergic receptor signaling in oligodendrocyte cells helps them in their development and for myelination, while in microglia purinergic receptors are known to function as immunocompetent. In all of these cases, ATP and other nucleotides work as danger signals by activating microglia in pathophysiological conditions. Importantly, dysregulations of purinoceptors have been associated with major CNS disorders including neurodegenerative diseases such as Alzheimer's disease (AD), Parkinson disease (PD), Amyotrophic lateral sclerosis (ALS), brain trauma, ischemia, epilepsy, and chronic pain associated with neuroinflammation, as well as neuropsychiatric diseases, including depression, anxiety, and schizophrenia. Importantly, the P2X_7_ receptor has been involved in all of the aforementioned diseases, and in many cases influenced by mutations that increase (or in some cases decrease) function, thus altering one's susceptibility for developing the disease. Moreover, P2X_7_ receptors have been shown to activate the NLRP3 inflammasome and the release of pro-inflammatory cytokines, which drives neuroinflammation, and recent work suggests that inhibition of these receptors may server as a viable drug target.

The role of P2X_7_ receptor in the production and release of active pro-inflammatory cytokine IL-1β has inspired major efforts to develop antagonists. A number of research groups have disclosed potent P2X_7_ receptor antagonists, which have been shown to attenuate the release of IL-1β from stimulated cells. Since P2X_7_ receptor is integral in the processing and release of many inflammatory mediators (IL-1β, NFκB, TNFα etc.), the inhibition of this receptor may provide therapeutic benefit in diseases including cancer, tuberculosis, diabetes, asthma, and all of the neurodegenerative diseases. While, full understanding of the P2X_7_R localization and function in the brain is currently incomplete, development of high potency and selective antagonists that cross the BBB have helped our understanding of the receptor in multiple rodent models of peripheral inflammatory diseases, neuroinflammatory disorders, and cancer. Although these data have begun to fill this knowledge gap, it is important to recognize that many of the rodent models are based on base-strains that retain SNPs, and therefore show dampened or reduced sensitivity to ATP and thus reduced signaling. Despite this, several antagonists have been studied in depression, anxiety, bipolar disorders, and PD. In some cases, clinical trials have been conducted to evaluate inflammatory pathologies such as rheumatoid arthritis, Crohn's, and basal cell carcinoma. The latest generation of antagonists have been designed to specifically penetrate brain and evaluate brain disorders. Several antagonists from this group have entered clinical trials for evaluation of the P2X_7_R participation in CNS disorders. In the future, the development of P2X_7_ receptor antagonist that are potent, selective, and have acceptable pharmacokinetic and pharmacodynamic readouts will be needed to advance the field.

## Author Contributions

PT and HZ contributed equally to the review article. Each author wrote various sections and provided extensive edits to the final document. PT generated the graphical abstract using online tools found at BioRender. All authors contributed to the article and approved the submitted version.

## Conflict of Interest

The authors declare that the research was conducted in the absence of any commercial or financial relationships that could be construed as a potential conflict of interest.

## References

[B1] Abdi M. H. Beswick P. J. Billinton A. Chambers L. J. Charlton A. Collins S. D.. (2010). Discovery and structure-activity relationships of a series of pyroglutamic acid amide antagonists of the P2X7 receptor. Bioorg. Med. Chem. Lett 20, 5080–5084. 10.1016/j.bmcl.2010.07.033 20673717

[B2] Adinolfi E. Giuliani A. L. De Marchi E. Pegoraro A. Orioli E. Di Virgilio F. (2018). The P2X7 receptor: a main player in inflammation. Biochem. Pharmacol 151, 234–244. 10.1016/j.bcp.2017.12.021 29288626

[B3] Ali Z. Laurijssens B. Ostenfeld T. Mchugh S. Stylianou A. Scott-Stevens P.. (2013). Pharmacokinetic and pharmacodynamic profiling of a P2X7 receptor allosteric modulator GSK1482160 in healthy human subjects. Br. J. Clin. Pharmacol. 75, 197–207. 10.1111/j.1365-2125.2012.04320.x 22568863 PMC3555059

[B4] Amadio S. Parisi C. Piras E. Fabbrizio P. Apolloni S. Montilli C.. (2017). Modulation of P2X7 receptor during inflammation in multiple sclerosis. Front. Immunol. 8:1529. 10.3389/fimmu.2017.01529 29187851 PMC5694754

[B5] Amhaoul H. Ali I. Mola M. Van Eetveldt A. Szewczyk K. Missault S.. (2016). P2X7 receptor antagonism reduces the severity of spontaneous seizures in a chronic model of temporal lobe epilepsy. Neuropharmacology 105, 175–185. 10.1016/j.neuropharm.2016.01.018 26775823

[B6] Apolloni S. Amadio S. Parisi C. Matteucci A. Potenza R. L. Armida M.. (2014). Spinal cord pathology is ameliorated by P2X7 antagonism in a SOD1-mutant mouse model of amyotrophic lateral sclerosis. Dis. Model. Mech. 7, 1101–1109. 10.1242/dmm.017038 25038061 PMC4142730

[B7] Apolloni S. Parisi C. Pesaresi M. G. Rossi S. Carri M. T. Cozzolino M.. (2013). The NADPH oxidase pathway is dysregulated by the P2X7 receptor in the SOD1-G93A microglia model of amyotrophic lateral sclerosis. J. Immunol 190, 5187–5195. 10.4049/jimmunol.1203262 23589615

[B8] Aprile-Garcia F. Metzger M. W. Paez-Pereda M. Stadler H. Acuna M. Liberman A. C.. (2016). Co-expression of wild-type P2X7R with Gln460Arg variant alters receptor function. PLoS ONE 11:e0151862. 10.1371/journal.pone.0151862 26986975 PMC4795689

[B9] Ballerini P. Rathbone M. P. Di Iorio P. Renzetti A. Giuliani P. D'alimonte I.. (1996). Rat astroglial P2Z (P2X7) receptors regulate intracellular calcium and purine release. Neuroreport 7, 2533–2537. 10.1097/00001756-199611040-00026 8981418

[B10] Barden N. Harvey M. Gagne B. Shink E. Tremblay M. Raymond C.. (2006). Analysis of single nucleotide polymorphisms in genes in the chromosome 12Q24.31 region points to P2RX7 as a susceptibility gene to bipolar affective disorder. Am. J. Med. Genet. B Neuropsychiatr. Genet 141B, 374–382. 10.1002/ajmg.b.30303 16673375

[B11] Bartlett R. Sluyter V. Watson D. Sluyter R. Yerbury J. J. (2017). P2X7 antagonism using Brilliant Blue G reduces body weight loss and prolongs survival in female SOD1(G93A) amyotrophic lateral sclerosis mice. PeerJ. 5:e3064. 10.7717/peerj.3064 28265522 PMC5335685

[B12] Bartlett R. Stokes L. Sluyter R. (2014). The P2X7 receptor channel: recent developments and the use of P2X7 antagonists in models of disease. Pharmacol. Rev 66, 638–675. 10.1124/pr.113.008003 24928329

[B13] Basso A. M. Bratcher N. A. Harris R. R. Jarvis M. F. Decker M. W. Rueter L. E. (2009). Behavioral profile of P2X7 receptor knockout mice in animal models of depression and anxiety: relevance for neuropsychiatric disorders. Behav. Brain Res. 198, 83–90. 10.1016/j.bbr.2008.10.018 18996151

[B14] Beaino W. Janssen B. Kooij G. Van Der Pol S. M. A. Van Het Hof B. Van Horssen J.. (2017). Purinergic receptors P2Y12R and P2X7R: potential targets for PET imaging of microglia phenotypes in multiple sclerosis. J. Neuroinflammation 14:259. 10.1186/s12974-017-1034-z 29273052 PMC5741931

[B15] Beamer E. Fischer W. Engel T. (2017). The ATP-Gated P2X7 Receptor As a Target for the Treatment of Drug-Resistant Epilepsy. Front. Neurosci. 11:21. 10.3389/fnins.2017.00021 28210205 PMC5288361

[B16] Beamer E. Goloncser F. Horvath G. Beko K. Otrokocsi L. Kovanyi B.. (2016). Purinergic mechanisms in neuroinflammation: An update from molecules to behavior. Neuropharmacology 104, 94–104. 10.1016/j.neuropharm.2015.09.019 26384652

[B17] Beigi R. D. Kertesy S. B. Aquilina G. Dubyak G. R. (2003). Oxidized ATP (oATP) attenuates proinflammatory signaling via P2 receptor-independent mechanisms. Br. J. Pharmacol. 140, 507–519. 10.1038/sj.bjp.0705470 14522842 PMC1574058

[B18] Bhattacharya A. (2018). Recent advances in CNS P2X7 physiology and pharmacology: focus on neuropsychiatric disorders. Front. Pharmacol. 9:30. 10.3389/fphar.2018.00030 29449810 PMC5799703

[B19] Bhattacharya A. Biber K. (2016). The microglial ATP-gated ion channel P2X7 as a CNS drug target. Glia 64, 1772–1787. 10.1002/glia.23001 27219534

[B20] Bhattacharya A. Jones D. N. C. (2018). Emerging role of the P2X7-NLRP3-IL1beta pathway in mood disorders. Psychoneuroendocrinology 98, 95–100. 10.1016/j.psyneuen.2018.08.015 30121550

[B21] Bhattacharya A. Lord B. Grigoleit J. S. He Y. Fraser I. Campbell S. N.. (2018). Neuropsychopharmacology of JNJ-55308942: evaluation of a clinical candidate targeting P2X7 ion channels in animal models of neuroinflammation and anhedonia. Neuropsychopharmacology 43, 2586–2596. 10.1038/s41386-018-0141-6 30026598 PMC6224414

[B22] Bhattacharya A. Wang Q. Ao H. Shoblock J. R. Lord B. Aluisio L.. (2013). Pharmacological characterization of a novel centrally permeable P2X7 receptor antagonist: JNJ-47965567. Br. J. Pharmacol. 170, 624–640. 10.1111/bph.12314 23889535 PMC3792000

[B23] Bianchi G. Vuerich M. Pellegatti P. Marimpietri D. Emionite L. Marigo I.. (2014). ATP/P2X7 axis modulates myeloid-derived suppressor cell functions in neuroblastoma microenvironment. Cell Death Dis. 5:e1135. 10.1038/cddis.2014.109 24651438 PMC3973218

[B24] Bin Dayel A. Evans R. J. Schmid R. (2019). Mapping the site of action of human P2X7 receptor antagonists AZ11645373, brilliant blue G, KN-62, calmidazolium, and ZINC58368839 to the intersubunit allosteric pocket. Mol. Pharmacol. 96, 355–363. 10.1124/mol.119.116715 31263019 PMC6701605

[B25] Boue-Grabot E. Pankratov Y. (2017). Modulation of central synapses by astrocyte-released ATP and postsynaptic P2X receptors. Neural Plast. 2017:9454275. 10.1155/2017/9454275 28845311 PMC5563405

[B26] Boumechache M. Masin M. Edwardson J. M. Gorecki D. C. Murrell-Lagnado R. (2009). Analysis of assembly and trafficking of native P2X4 and P2X7 receptor complexes in rodent immune cells. J. Biol. Chem 284, 13446–13454. 10.1074/jbc.M901255200 19304656 PMC2679444

[B27] Braun N. Sevigny J. Robson S. C. Enjyoji K. Guckelberger O. Hammer K.. (2000). Assignment of ecto-nucleoside triphosphate diphosphohydrolase-1/cd39 expression to microglia and vasculature of the brain. Eur. J. Neurosci 12, 4357–4366. 10.1046/j.1460-9568.2000.01342.x 11122346

[B28] Broom D. C. Matson D. J. Bradshaw E. Buck M. E. Meade R. Coombs S.. (2008). Characterization of N-(adamantan-1-ylmethyl)-5-[(3R-amino-pyrrolidin-1-yl)methyl]-2-chloro-benzamide, a P2X7 antagonist in animal models of pain and inflammation. J. Pharmacol. Exp. Ther 327, 620–633. 10.1124/jpet.108.141853 18772321

[B29] Burnstock G. (2006a). Historical review: ATP as a neurotransmitter. Trends Pharmacol. Sci 27, 166–176. 10.1016/j.tips.2006.01.005 16487603

[B30] Burnstock G. (2006b). Pathophysiology and therapeutic potential of purinergic signaling. Pharmacol. Rev 58, 58–86. 10.1124/pr.58.1.5 16507883

[B31] Burnstock G. (2007). Physiology and pathophysiology of purinergic neurotransmission. Physiol. Rev 87, 659–797. 10.1152/physrev.00043.2006 17429044

[B32] Burnstock G. (2008). Purinergic signalling and disorders of the central nervous system. Nat. Rev. Drug Discov 7, 575–590. 10.1038/nrd2605 18591979

[B33] Burnstock G. (2015). Purinergic signalling in neuroregeneration. Neural Regen Res 10, 1919. 10.4103/1673-5374.165300 26889167 PMC4730803

[B34] Burnstock G. (2016). An introduction to the roles of purinergic signalling in neurodegeneration, neuroprotection and neuroregeneration. Neuropharmacology 104, 4–17. 10.1016/j.neuropharm.2015.05.031 26056033

[B35] Burnstock G. (2018). Purine and purinergic receptors. Brain Neurosci. Adv. 2, 1–10. 10.1177/2398212818817494 32166165 PMC7058212

[B36] Burnstock G. Krugel U. Abbracchio M. P. Illes P. (2011). Purinergic signalling: from normal behaviour to pathological brain function. Prog Neurobiol. 95, 229–274. 10.1016/j.pneurobio.2011.08.006 21907261

[B37] Carmo M. R. Menezes A. P. Nunes A. C. Pliassova A. Rolo A. P. Palmeira C. M.. (2014). The P2X7 receptor antagonist Brilliant Blue G attenuates contralateral rotations in a rat model of Parkinsonism through a combined control of synaptotoxicity, neurotoxicity and gliosis. Neuropharmacology 81, 142–152. 10.1016/j.neuropharm.2014.01.045 24508709

[B38] Caseley E. A. Muench S. P. Roger S. Mao H. J. Baldwin S. A. Jiang L. H. (2014). Non-synonymous single nucleotide polymorphisms in the P2X receptor genes: association with diseases, impact on receptor functions and potential use as diagnosis biomarkers. Int. J. Mol. Sci 15, 13344–13371. 10.3390/ijms150813344 25079442 PMC4159798

[B39] Catanzaro J. M. Hueston C. M. Deak M. M. Deak T. (2014). The impact of the P2X7 receptor antagonist A-804598 on neuroimmune and behavioral consequences of stress. Behav. Pharmacol 25, 582–598. 10.1097/FBP.0000000000000072 25083574

[B40] Cctu-Core (2019). Antidepressant Trial With P2X7 Antagonist JNJ-54175446 (ATP) [Online]. National Institutes of Health. Available online at: https://clinicaltrials.gov/ct2/show/NCT04116606 (accessed May 10, 2020).

[B41] Chang B. S. Lowenstein D. H. (2003). Epilepsy. N. Engl. J. Med 349, 1257–1266. 10.1056/NEJMra022308 14507951

[B42] Chen S. Ma Q. Krafft P. R. Chen Y. Tang J. Zhang J.. (2013a). P2X7 receptor antagonism inhibits p38 mitogen-activated protein kinase activation and ameliorates neuronal apoptosis after subarachnoid hemorrhage in rats. Crit. Care Med. 41, e466–474. 10.1097/CCM.0b013e31829a8246 23963136 PMC3841260

[B43] Chen S. Ma Q. Krafft P. R. Hu Q. Rolland W. 2nd Sherchan P. Zhang J.. (2013b). P2X7R/cryopyrin inflammasome axis inhibition reduces neuroinflammation after SAH. Neurobiol. Dis. 58, 296–307. 10.1016/j.nbd.2013.06.011 23816751 PMC3771387

[B44] Chen X. Hu J. Jiang L. Xu S. Zheng B. Wang C.. (2014). Brilliant Blue G improves cognition in an animal model of Alzheimer's disease and inhibits amyloid-beta-induced loss of filopodia and dendrite spines in hippocampal neurons. Neuroscience 279, 94–101. 10.1016/j.neuroscience.2014.08.036 25193238

[B45] Choi I. S. Cho J. H. Lee M. G. Jang I. S. (2015). Enzymatic conversion of ATP to adenosine contributes to ATP-induced inhibition of glutamate release in rat medullary dorsal horn neurons. Neuropharmacology 93, 94–102. 10.1016/j.neuropharm.2015.01.020 25656480

[B46] Chrovian C. C. Soyode-Johnson A. Peterson A. A. Gelin C. F. Deng X. Dvorak C. A.. (2018). A dipolar cycloaddition reaction to access 6-Methyl-4,5,6,7-tetrahydro-1H-[1,2,3]triazolo[4,5-c]pyridines enables the discovery synthesis and preclinical profiling of a P2X7 antagonist clinical candidate. J. Med. Chem. 61, 207–223. 10.1021/acs.jmedchem.7b01279 29211470

[B47] Chu K. Yin B. Wang J. Peng G. Liang H. Xu Z.. (2012). Inhibition of P2X7 receptor ameliorates transient global cerebral ischemia/reperfusion injury via modulating inflammatory responses in the rat hippocampus. J. Neuroinflammation 9:69. 10.1186/1742-2094-9-69 22513224 PMC3418181

[B48] Cieslak M. Wojtczak A. Komoszynski M. (2017). Role of the purinergic signaling in epilepsy. Pharmacol. Rep. 69, 130–138. 10.1016/j.pharep.2016.09.018 27915186

[B49] Cunha R. A. (2016). How does adenosine control neuronal dysfunction and neurodegeneration? J. Neurochem. 139, 1019–1055. 10.1111/jnc.13724 27365148

[B50] D'ambrosi N. Finocchi P. Apolloni S. Cozzolino M. Ferri A. Padovano V.. (2009). The proinflammatory action of microglial P2 receptors is enhanced in SOD1 models for amyotrophic lateral sclerosis. J. Immunol. 183, 4648–4656. 10.4049/jimmunol.0901212 19734218

[B51] De Marchi E. Orioli E. Dal Ben D. Adinolfi E. (2016). P2X7 receptor as a therapeutic target. Adv. Protein Chem. Struct. Biol 104, 39–79. 10.1016/bs.apcsb.2015.11.004 27038372

[B52] Delarasse C. Auger R. Gonnord P. Fontaine B. Kanellopoulos J. M. (2011). The purinergic receptor P2X7 triggers alpha-secretase-dependent processing of the amyloid precursor protein. J. Biol. Chem. 286, 2596–2606. 10.1074/jbc.M110.200618 21081501 PMC3024755

[B53] Di Virgilio F. (2003). Novel data point to a broader mechanism of action of oxidized ATP: the P2X7 receptor is not the only target. Br. J. Pharmacol. 140, 441–443. 10.1038/sj.bjp.0705469 14522840 PMC1574057

[B54] Di Virgilio F. (2020). P2X7 is a cytotoxic receptor maybe not: implications for cancer. Purinergic Signal. 10.1007/s11302-020-09735-w 33011962 PMC7955003

[B55] Di Virgilio F. Ceruti S. Bramanti P. Abbracchio M. P. (2009). Purinergic signalling in inflammation of the central nervous system. Trends Neurosci. 32, 79–87. 10.1016/j.tins.2008.11.003 19135728

[B56] Di Virgilio F. Chiozzi P. Ferrari D. Falzoni S. Sanz J. M. Morelli A.. (2001). Nucleotide receptors: an emerging family of regulatory molecules in blood cells. Blood 97, 587–600. 10.1182/blood.V97.3.587 11157473

[B57] Di Virgilio F. Dal Ben D. Sarti A. C. Giuliani A. L. Falzoni S. (2017). The P2X7 receptor in infection and inflammation. Immunity 47, 15–31. 10.1016/j.immuni.2017.06.020 28723547

[B58] Di Virgilio F. Schmalzing G. Markwardt F. (2018). The elusive P2X7 macropore. Trends Cell Biol. 28, 392–404. 10.1016/j.tcb.2018.01.005 29439897

[B59] Diaz-Hernandez J. I. Gomez-Villafuertes R. Leon-Otegui M. Hontecillas-Prieto L. Del Puerto A. Trejo J. L.. (2012). *In vivo* P2X7 inhibition reduces amyloid plaques in Alzheimer's disease through GSK3beta and secretases. Neurobiol. Aging 33, 1816–1828. 10.1016/j.neurobiolaging.2011.09.040 22048123

[B60] Diaz-Hernandez M. Diez-Zaera M. Sanchez-Nogueiro J. Gomez-Villafuertes R. Canals J. M. Alberch J.. (2009). Altered P2X7-receptor level and function in mouse models of Huntington's disease and therapeutic efficacy of antagonist administration. FASEB J 23, 1893–1906. 10.1096/fj.08-122275 19171786

[B61] Domercq M. Perez-Samartin A. Aparicio D. Alberdi E. Pampliega O. Matute C. (2010). P2X7 receptors mediate ischemic damage to oligodendrocytes. Glia 58, 730–740. 10.1002/glia.20958 20029962

[B62] Domercq M. Vazquez-Villoldo N. Matute C. (2013). Neurotransmitter signaling in the pathophysiology of microglia. Front. Cell. Neurosci 7:49. 10.3389/fncel.2013.00049 23626522 PMC3630369

[B63] Donnelly-Roberts D. L. Namovic M. T. Han P. Jarvis M. F. (2009). Mammalian P2X7 receptor pharmacology: comparison of recombinant mouse, rat and human P2X7 receptors. Br. J. Pharmacol. 157, 1203–1214. 10.1111/j.1476-5381.2009.00233.x 19558545 PMC2743839

[B64] Durrenberger P. F. Grünblatt E. Fernando F. S. Monoranu C. M. Evans J. Riederer P.. (2012). Inflammatory Pathways in Parkinson's Disease: a BNE microarray study. Parkinson's Dis. 2012:214714. 10.1155/2012/214714 22548201 PMC3324922

[B65] Engel T. Alves M. Sheedy C. Henshall D. C. (2016). ATPergic signalling during seizures and epilepsy. Neuropharmacology 104, 140–153. 10.1016/j.neuropharm.2015.11.001 26549853

[B66] Evotec (2020). Evotec Announces the Successful Completion of the First Phase I Study with EVT 401, an Oral P2X7 Receptor Antagonist - Very Good Safety Profile and Confirmed “On Target Activity” [Online]. Hamburg: Evotec.

[B67] Eyo U. B. Miner S. A. Ahlers K. E. Wu L. J. Dailey M. E. (2013). P2X7 receptor activation regulates microglial cell death during oxygen-glucose deprivation. Neuropharmacology 73, 311–319. 10.1016/j.neuropharm.2013.05.032 23770338 PMC3786777

[B68] Faas M. M. Saez T. De Vos P. (2017). Extracellular ATP and adenosine: the Yin and Yang in immune responses? Mol. Aspects Med. 55, 9–19. 10.1016/j.mam.2017.01.002 28093236

[B69] Fabbrizio P. Amadio S. Apolloni S. Volonte C. (2017). P2X7 receptor activation modulates autophagy in SOD1-G93A mouse microglia. Front. Cell. Neurosci. 11:249. 10.3389/fncel.2017.00249 28871219 PMC5566572

[B70] Faloia E. Michetti G. Grazia M. De Robertis M. Luconi M. P. Furlani G.. (2012). Inflammation as a link between obesity and metabolic syndrome. J. Nutr. Metab. 2012:476380. 10.1155/2012/476380 22523672 PMC3317136

[B71] Felix R. A. Martin S. Pinion S. Crawford D. J. (2012). Development of a comprehensive set of P2 receptor pharmacological research compounds. Purinergic Signal 8, 101–112. 10.1007/s11302-011-9270-7 22052555 PMC3265712

[B72] Feng L. Chen Y. Ding R. Fu Z. Yang S. Deng X.. (2015). P2X7R blockade prevents NLRP3 inflammasome activation and brain injury in a rat model of intracerebral hemorrhage: involvement of peroxynitrite. J. Neuroinflammation 12:190. 10.1186/s12974-015-0409-2 26475134 PMC4609067

[B73] Ferrazoli E. G. De Souza H. D. Nascimento I. C. Oliveira-Giacomelli A. Schwindt T. T. Britto L. R.. (2017). Brilliant Blue G, but not fenofibrate, treatment reverts hemiparkinsonian behavior and restores dopamine levels in an animal model of Parkinson's Disease. Cell Transplant 26, 669–677. 10.3727/096368917X695227 28403913 PMC5661224

[B74] Fiebich B. L. Akter S. Akundi R. S. (2014). The two-hit hypothesis for neuroinflammation: role of exogenous ATP in modulating inflammation in the brain. Front. Cell. Neurosci. 8:260. 10.3389/fncel.2014.00260 25225473 PMC4150257

[B75] Fischer W. Franke H. Krugel U. Muller H. Dinkel K. Lord B.. (2016). Critical evaluation of P2X7 receptor antagonists in selected seizure models. PLoS ONE 11:e0156468. 10.1371/journal.pone.0156468 27281030 PMC4900628

[B76] Florjancic A. S. Peddi S. Perez-Medrano A. Li B. Namovic M. T. Grayson G.. (2008). Synthesis and *in vitro* activity of 1-(2,3-dichlorophenyl)-N-(pyridin-3-ylmethyl)-1H-1,2,4-triazol-5-amine and 4-(2,3-dichlorophenyl)-N-(pyridin-3-ylmethyl)-4H-1,2,4-triazol-3-amine P2X7 antagonists. Bioorg. Med. Chem. Lett. 18, 2089–2092. 10.1016/j.bmcl.2008.01.095 18272366

[B77] Franke H. Verkhratsky A. Burnstock G. Illes P. (2012). Pathophysiology of astroglial purinergic signalling. Purinergic Signal 8, 629–657. 10.1007/s11302-012-9300-0 22544529 PMC3360100

[B78] Franklin T. C. Xu C. Duman R. S. (2018). Depression and sterile inflammation: essential role of danger associated molecular patterns. Brain Behav. Immun. 72, 2–13. 10.1016/j.bbi.2017.10.025 29102801

[B79] Fredholm B. B. Ap I. J. Jacobson K. A. Klotz K. N. Linden J. (2001). International Union of Pharmacology. XXV. Nomenclature and classification of adenosine receptors. Pharmacol. Rev. 53, 527–552. 11734617 PMC9389454

[B80] Freire D. Reyes R. E. Baghram A. Davies D. L. Asatryan L. (2019). P2X7 receptor antagonist A804598 inhibits inflammation in brain and liver in C57BL/6J mice exposed to chronic ethanol and high fat diet. J. Neuroimmune Pharmacol. 14, 263–277. 10.1007/s11481-018-9816-3 30353422 PMC6494709

[B81] Friedle S. A. Curet M. A. Watters J. J. (2010). Recent patents on novel P2X(7) receptor antagonists and their potential for reducing central nervous system inflammation. Recent Pat. CNS Drug Discov 5, 35–45. 10.2174/157488910789753530 19705995 PMC2794961

[B82] Gandelman M. Peluffo H. Beckman J. S. Cassina P. Barbeito L. (2010). Extracellular ATP and the P2X7 receptor in astrocyte-mediated motor neuron death: implications for amyotrophic lateral sclerosis. J. Neuroinflammation 7:33. 10.1186/1742-2094-7-33 20534165 PMC2901222

[B83] Gao M. Wang M. Glick-Wilson B. E. Meyer J. A. Peters J. S. Territo P. R.. (2018). Synthesis and preliminary biological evaluation of a novel P2X7R radioligand [(18)F]IUR-1601. Bioorg. Med. Chem. Lett 28, 1603–1609. 10.1016/j.bmcl.2018.03.044 29628324

[B84] Gever J. R. Cockayne D. A. Dillon M. P. Burnstock G. Ford A. P. (2006). Pharmacology of P2X channels. Pflugers Arch 452, 513–537. 10.1007/s00424-006-0070-9 16649055

[B85] Ghiringhelli F. Apetoh L. Tesniere A. Aymeric L. Ma Y. Ortiz C.. (2009). Activation of the NLRP3 inflammasome in dendritic cells induces IL-1beta-dependent adaptive immunity against tumors. Nat. Med. 15, 1170–1178. 10.1038/nm.2028 19767732

[B86] Glaxosmithkline (2009). First Time in Human Study Evaluating the Safety, Tolerability, Pharmacokinetics, Pharmacodynamics and the Effect of Food of Single Assending Doses of GSK1482160 [Online]. Bethesda, MD: National Institutes of Health.

[B87] Grygorowicz T. Struzynska L. Sulkowski G. Chalimoniuk M. Sulejczak D. (2010). Temporal expression of P2X7 purinergic receptor during the course of experimental autoimmune encephalomyelitis. Neurochem. Int. 57, 823–829. 10.1016/j.neuint.2010.08.021 20817062

[B88] Grygorowicz T. Welniak-Kaminska M. Struzynska L. (2016). Early P2X7R-related astrogliosis in autoimmune encephalomyelitis. Mol. Cell. Neurosci. 74, 1–9. 10.1016/j.mcn.2016.02.003 26921791

[B89] Gu B. J. Field J. Dutertre S. Ou A. Kilpatrick T. J. Lechner-Scott J.. (2015). A rare P2X7 variant Arg307Gln with absent pore formation function protects against neuroinflammation in multiple sclerosis. Hum. Mol. Genet. 24, 5644–5654. 10.1093/hmg/ddv278 26188005

[B90] Gu B. J. Zhang W. Worthington R. A. Sluyter R. Dao-Ung P. Petrou S.. (2001). A Glu-496 to Ala polymorphism leads to loss of function of the human P2X7 receptor. J. Biol. Chem. 276, 11135–11142. 10.1074/jbc.M010353200 11150303

[B91] Guo C. Masin M. Qureshi O. S. Murrell-Lagnado R. D. (2007). Evidence for functional P2X4/P2X7 heteromeric receptors. Mol. Pharmacol. 72, 1447–1456. 10.1124/mol.107.035980 17785580

[B92] Hagens M. H. J. Golla S. S. V. Janssen B. Vugts D. J. Beaino W. Windhorst A. D.. (2020). The P2X7 receptor tracer [(11)C]SMW139 as an *in vivo* marker of neuroinflammation in multiple sclerosis: a first-in man study. Eur. J. Nucl. Med. Mol. Imaging 47, 379–389. 10.1007/s00259-019-04550-x 31705174 PMC6974509

[B93] Hansen T. Bubb K. Kassiou M. Figtree G. (2018). Abstract 17368: the novel P2X7 receptor antagonist SMW139 inhibits inflammasome activation in STEMI monocytes. Circulation. 138:A17368.

[B94] Haynes S. E. Hollopeter G. Yang G. Kurpius D. Dailey M. E. Gan W. B.. (2006). The P2Y12 receptor regulates microglial activation by extracellular nucleotides. Nat. Neurosci. 9, 1512–1519. 10.1038/nn1805 17115040

[B95] He Y. Taylor N. Fourgeaud L. Bhattacharya A. (2017). The role of microglial P2X7: modulation of cell death and cytokine release. J. Neuroinflammation 14:135. 10.1186/s12974-017-0904-8 28716092 PMC5513370

[B96] Hempel C. Norenberg W. Sobottka H. Urban N. Nicke A. Fischer W.. (2013). The phenothiazine-class antipsychotic drugs prochlorperazine and trifluoperazine are potent allosteric modulators of the human P2X7 receptor. Neuropharmacology 75, 365–379. 10.1016/j.neuropharm.2013.07.027 23954492

[B97] Henshall D. C. Diaz-Hernandez M. Miras-Portugal M. T. Engel T. (2013). P2X receptors as targets for the treatment of status epilepticus. Front. Cell. Neurosci 7, 237. 10.3389/fncel.2013.00237 24324404 PMC3840793

[B98] Honore P. Donnelly-Roberts D. Namovic M. Zhong C. Wade C. Chandran P.. (2009). The antihyperalgesic activity of a selective P2X7 receptor antagonist, A-839977, is lost in IL-1alphabeta knockout mice. Behav. Brain Res. 204, 77–81. 10.1016/j.bbr.2009.05.018 19464323

[B99] Honore P. Donnelly-Roberts D. Namovic M. T. Hsieh G. Zhu C. Z. Mikusa J. P.. (2006). A-740003 [N-(1-{[(cyanoimino)(5-quinolinylamino) methyl]amino}-2,2-dimethylpropyl)-2-(3,4-dimethoxyphenyl)acetamide], a novel and selective P2X7 receptor antagonist, dose-dependently reduces neuropathic pain in the rat. J. Pharmacol. Exp. Ther. 319, 1376–1385. 10.1124/jpet.106.111559 16982702

[B100] Hospital T. U. (2019). Multimodal Imaging of MS Reveals the Smoldering Inflammation (PLAQ-MS) [Online]. Bethesda MA: National Institues of Health.

[B101] Hracsko Z. Baranyi M. Csolle C. Goloncser F. Madarasz E. Kittel A.. (2011). Lack of neuroprotection in the absence of P2X7 receptors in toxin-induced animal models of Parkinson's disease. Mol. Neurodegener. 6:28. 10.1186/1750-1326-6-28 21542899 PMC3113297

[B102] Idzko M. Ferrari D. Riegel A. K. Eltzschig H. K. (2014). Extracellular nucleotide and nucleoside signaling in vascular and blood disease. Blood 124, 1029–1037. 10.1182/blood-2013-09-402560 25001468 PMC4133480

[B103] Illes P. Khan T. M. Rubini P. (2017). Neuronal P2X7 receptors revisited: do they really exist? J. Neurosci. 37, 7049–7062. 10.1523/JNEUROSCI.3103-16.2017 28747388 PMC6705732

[B104] Inoue K. (2008). Purinergic systems in microglia. Cell. Mol. Life Sci 65, 3074–3080. 10.1007/s00018-008-8210-3 18563292 PMC11131657

[B105] Jacobson K. A. Muller C. E. (2016). Medicinal chemistry of adenosine, P2Y and P2X receptors. Neuropharmacology 104, 31–49. 10.1016/j.neuropharm.2015.12.001 26686393 PMC4871727

[B106] Janssen B. Vugts D. J. Funke U. Spaans A. Schuit R. C. Kooijman E.. (2014). Synthesis and initial preclinical evaluation of the P2X7 receptor antagonist [(1)(1)C]A-740003 as a novel tracer of neuroinflammation. J. Labelled Comp. Radiopharm. 57, 509–516. 10.1002/jlcr.3206 24995673

[B107] Janssen B. Vugts D. J. Wilkinson S. M. Ory D. Chalon S. Hoozemans J. J. M.. (2018). Identification of the allosteric P2X7 receptor antagonist [(11)C]SMW139 as a PET tracer of microglial activation. Sci. Rep 8:6580. 10.1038/s41598-018-24814-0 29700413 PMC5920098

[B108] Jiang L.-H. (2012). P2X receptor-mediated ATP purinergic signaling in health and disease. Cell Health Cytoskelet. 4, 83–101. 10.2147/CHC.S27196

[B109] Jiang L.-H. Mackenzie A. B. North R. A. Surprenant A. (2000). Brilliant blue G selectively blocks ATP-gated rat P2X7 receptors. Mol. Pharmacol 58 82–88. 10.1124/mol.58.1.82 10860929

[B110] Jiang L. H. (2009). Inhibition of P2X(7) receptors by divalent cations: old action and new insight. Eur. Biophys. J 38, 339–346. 10.1007/s00249-008-0315-y 18414844

[B111] Jiang L. H. Baldwin J. M. Roger S. Baldwin S. A. (2013). Insights into the molecular mechanisms underlying mammalian P2X7 receptor functions and contributions in diseases, revealed by structural modeling and single nucleotide polymorphisms. Front. Pharmacol. 4:55. 10.3389/fphar.2013.00055 23675347 PMC3646254

[B112] Jiang T. Hoekstra J. Heng X. Kang W. Ding J. Liu J.. (2015). P2X7 receptor is critical in alpha-synuclein–mediated microglial NADPH oxidase activation. Neurobiol. Aging 36, 2304–2318. 10.1016/j.neurobiolaging.2015.03.015 25983062

[B113] Jimenez-Pacheco A. Diaz-Hernandez M. Arribas-Blazquez M. Sanz-Rodriguez A. Olivos-Ore L. A. Artalejo A. R.. (2016). Transient P2X7 receptor antagonism produces lasting reductions in spontaneous seizures and gliosis in experimental temporal lobe epilepsy. J. Neurosci. 36, 5920–5932. 10.1523/JNEUROSCI.4009-15.2016 27251615 PMC6601816

[B114] Jo S. Bean B. P. (2011). Inhibition of neuronal voltage-gated sodium channels by brilliant blue G. Mol. Pharmacol. 80, 247–257. 10.1124/mol.110.070276 21536754 PMC3141889

[B115] Kanellopoulos J. M. Delarasse C. (2019). Pleiotropic roles of P2X7 in the central nervous system. Front. Cell. Neurosci. 13:401. 10.3389/fncel.2019.00401 31551714 PMC6738027

[B116] Karasawa A. Kawate T. (2016). Structural basis for subtype-specific inhibition of the P2X7 receptor. Elife 5:25. 10.7554/eLife.22153.025 27935479 PMC5176352

[B117] Keystone E. C. Wang M. M. Layton M. Hollis S. Mcinnes I. B. Team D. C. S. (2012). Clinical evaluation of the efficacy of the P2X7 purinergic receptor antagonist AZD9056 on the signs and symptoms of rheumatoid arthritis in patients with active disease despite treatment with methotrexate or sulphasalazine. Ann. Rheum. Dis. 71, 1630–1635. 10.1136/annrheumdis-2011-143578 22966146

[B118] Kim M. Jiang L. H. Wilson H. L. North R. A. Surprenant A. (2001). Proteomic and functional evidence for a P2X7 receptor signalling complex. EMBO J. 20, 6347–6358. 10.1093/emboj/20.22.6347 11707406 PMC125721

[B119] Kimbler D. E. Shields J. Yanasak N. Vender J. R. Dhandapani K. M. (2012). Activation of P2X7 promotes cerebral edema and neurological injury after traumatic brain injury in mice. PLoS ONE 7:e41229. 10.1371/journal.pone.0041229 22815977 PMC3398891

[B120] Kolb H. C. Barret O. Bhattacharya A. Chen G. Constantinescu C. Huang C.. (2019). Preclinical evaluation and nonhuman primate receptor occupancy study of (18)F-JNJ-64413739, a PET radioligand for P2X7 receptors. J. Nucl. Med. 60, 1154–1159. 10.2967/jnumed.118.212696 30733317

[B121] Koole M. Schmidt M. E. Hijzen A. Ravenstijn P. Vandermeulen C. Van Weehaeghe D.. (2019). (18)F-JNJ-64413739, a novel PET ligand for the P2X7 ion channel: radiation dosimetry, kinetic modeling, test-retest variability, and occupancy of the P2X7 antagonist JNJ-54175446. J. Nucl. Med. 60, 683–690. 10.2967/jnumed.118.216747 30262518

[B122] Kopp R. Krautloher A. Ramirez-Fernandez A. Nicke A. (2019). P2X7 interactions and signaling - making head or tail of it. Front. Mol. Neurosci 12:183. 10.3389/fnmol.2019.00183 31440138 PMC6693442

[B123] Kumar S. Mishra A. Krishnamurthy S. (2017). Purinergic antagonism prevents mitochondrial dysfunction and behavioral deficits associated with dopaminergic toxicity induced by 6-OHDA in rats. Neurochem. Res 42, 3414–3430. 10.1007/s11064-017-2383-9 28836128

[B124] Leeson H. C. Chan-Ling T. Lovelace M. D. Brownlie J. C. Gu B. J. Weible M. W. (2019). P2X7 receptor signaling during adult hippocampal neurogenesis. Neural Regen Res. 14, 1684–1694. 10.4103/1673-5374.257510 31169175 PMC6585562

[B125] Leff P. Wood B. E. O'connor S. E. (1990). Suramin is a slowly-equilibrating but competitive antagonist at P2x-receptors in the rabbit isolated ear artery. Br. J. Pharmacol. 101, 645–649. 10.1111/j.1476-5381.1990.tb14134.x 2076483 PMC1917720

[B126] Letavic M. A. Savall B. M. Allison B. D. Aluisio L. Andres J. I. De Angelis M.. (2017). 4-Methyl-6,7-dihydro-4H-triazolo[4,5-c]pyridine-based P2X7 receptor antagonists: optimization of pharmacokinetic properties leading to the identification of a clinical candidate. J. Med. Chem. 60, 4559–4572. 10.1021/acs.jmedchem.7b00408 28493698

[B127] Li Q. Barres B. A. (2018). Microglia and macrophages in brain homeostasis and disease. Nat. Rev. Immunol. 18, 225–242. 10.1038/nri.2017.125 29151590

[B128] Liang L. Schwiebert E. M. (2005). Large pore formation uniquely associated with P2X7 purinergic receptor channels. Focus on “Are second messengers crucial for opening the pore associated with P2X7 receptor?” Am J Physiol Cell Physiol. 288, C240–242. 10.1152/ajpcell.00532.2004 15643049

[B129] Liu F. Q. Gao Q. Wang D. D. Zhang Z. X. (2018). Effects of GBE50 on LPS/ATP induced NLRP3 inflammasome activation in primary rat microglia. Zhongguo Zhong Yao Za Zhi 43, 3346–3352. 30200740 10.19540/j.cnki.cjcmm.20180504.001

[B130] Liu J. Prell T. Stubendorff B. Keiner S. Ringer T. Gunkel A.. (2016). Down-regulation of purinergic P2X7 receptor expression and intracellular calcium dysregulation in peripheral blood mononuclear cells of patients with amyotrophic lateral sclerosis. Neurosci. Lett. 630, 77–83. 10.1016/j.neulet.2016.07.039 27453058

[B131] Lord B. Aluisio L. Shoblock J. R. Neff R. A. Varlinskaya E. I. Ceusters M.. (2014). Pharmacology of a novel central nervous system-penetrant P2X7 antagonist JNJ-42253432. J. Pharmacol. Exp. Ther. 351, 628–641. 10.1124/jpet.114.218487 25271258

[B132] Lord B. Ameriks M. K. Wang Q. Fourgeaud L. Vliegen M. Verluyten W.. (2015). A novel radioligand for the ATP-gated ion channel P2X7: [3H] JNJ-54232334. Eur. J. Pharmacol. 765, 551–559. 10.1016/j.ejphar.2015.09.026 26386289

[B133] Lucae S. Salyakina D. Barden N. Harvey M. Gagne B. Labbe M.. (2006). P2RX7, a gene coding for a purinergic ligand-gated ion channel, is associated with major depressive disorder. Hum. Mol. Genet. 15, 2438–2445. 10.1093/hmg/ddl166 16822851

[B134] Ly D. Dongol A. Cuthbertson P. Guy T. V. Geraghty N. J. Sophocleous R. A.. (2020). The P2X7 receptor antagonist JNJ-47965567 administered thrice weekly from disease onset does not alter progression of amyotrophic lateral sclerosis in SOD1(G93A) mice. Purinergic Signal 16, 109–122. 10.1007/s11302-020-09692-4 32170537 PMC7166237

[B135] Marcellino D. Suarez-Boomgaard D. Sanchez-Reina M. D. Aguirre J. A. Yoshitake T. Yoshitake S.. (2010). On the role of P2X(7) receptors in dopamine nerve cell degeneration in a rat model of Parkinson's disease: studies with the P2X(7) receptor antagonist A-438079. J. Neural. Transm. 117, 681–687. 10.1007/s00702-010-0400-0 20387084

[B136] Martin E. Amar M. Dalle C. Youssef I. Boucher C. Le Duigou C.. (2019). New role of P2X7 receptor in an Alzheimer's disease mouse model. Mol. Psychiatry 24, 108–125. 10.1038/s41380-018-0108-3 29934546 PMC6756107

[B137] Matute C. Torre I. Perez-Cerda F. Perez-Samartin A. Alberdi E. Etxebarria E.. (2007). P2X(7) receptor blockade prevents ATP excitotoxicity in oligodendrocytes and ameliorates experimental autoimmune encephalomyelitis. J. Neurosci. 27, 9525–9533. 10.1523/JNEUROSCI.0579-07.2007 17728465 PMC6673129

[B138] Mclarnon J. G. Ryu J. K. Walker D. G. Choi H. B. (2006). Upregulated expression of purinergic P2X(7) receptor in Alzheimer disease and amyloid-beta peptide-treated microglia and in peptide-injected rat hippocampus. J. Neuropathol. Exp. Neurol. 65, 1090–1097. 10.1097/01.jnen.0000240470.97295.d3 17086106

[B139] Mehta N. Kaur M. Singh M. Chand S. Vyas B. Silakari P.. (2014). Purinergic receptor P2X(7): a novel target for anti-inflammatory therapy. Bioorg. Med. Chem. 22, 54–88. 10.1016/j.bmc.2013.10.054 24314880

[B140] Melani A. Amadio S. Gianfriddo M. Vannucchi M. G. Volonte C. Bernardi G.. (2006). P2X7 receptor modulation on microglial cells and reduction of brain infarct caused by middle cerebral artery occlusion in rat. J. Cereb. Blood Flow Metab. 26, 974–982. 10.1038/sj.jcbfm.9600250 16395292

[B141] Mesuret G. Engel T. Hessel E. V. Sanz-Rodriguez A. Jimenez-Pacheco A. Miras-Portugal M. T.. (2014). P2X7 receptor inhibition interrupts the progression of seizures in immature rats and reduces hippocampal damage. CNS Neurosci. Ther. 20, 556–564. 10.1111/cns.12272 24750893 PMC6493144

[B142] Metzger M. W. Walser S. M. Dedic N. Aprile-Garcia F. Jakubcakova V. Adamczyk M.. (2017). Heterozygosity for the mood disorder-associated variant Gln460Arg alters P2X7 receptor function and sleep quality. J. Neurosci. 37, 11688–11700. 10.1523/JNEUROSCI.3487-16.2017 29079688 PMC6705750

[B143] Michel A. D. Chambers L. J. Clay W. C. Condreay J. P. Walter D. S. Chessell I. P. (2007). Direct labelling of the human P2X7 receptor and identification of positive and negative cooperativity of binding. Br. J. Pharmacol. 151, 103–114. 10.1038/sj.bjp.0707196 17339830 PMC2012979

[B144] Miller C. M. Boulter N. R. Fuller S. J. Zakrzewski A. M. Lees M. P. Saunders B. M.. (2011). The role of the P2X(7) receptor in infectious diseases. PLoS Pathog. 7:e1002212. 10.1371/journal.ppat.1002212 22102807 PMC3213081

[B145] Miras-Portugal M. T. Diaz-Hernandez M. Giraldez L. Hervas C. Gomez-Villafuertes R. Sen R. P.. (2003). P2X7 receptors in rat brain: presence in synaptic terminals and granule cells. Neurochem. Res. 28, 1597–1605. 10.1023/A:1025690913206 14570406

[B146] Monif M. Burnstock G. Williams D. A. (2010). Microglia: proliferation and activation driven by the P2X7 receptor. Int. J. Biochem. Cell Biol. 42, 1753–1756. 10.1016/j.biocel.2010.06.021 20599520

[B147] Murrell-Lagnado R. D. (2017). Regulation of P2X purinergic receptor signaling by cholesterol. Curr. Top. Membr. 80, 211–232. 10.1016/bs.ctm.2017.05.004 28863817

[B148] Narcisse L. Scemes E. Zhao Y. Lee S. C. Brosnan C. F. (2005). The cytokine IL-1beta transiently enhances P2X7 receptor expression and function in human astrocytes. Glia 49, 245–258. 10.1002/glia.20110 15472991 PMC2586293

[B149] Nelson D. W. Gregg R. J. Kort M. E. Perez-Medrano A. Voight E. A. Wang Y.. (2006). Structure-activity relationship studies on a series of novel, substituted 1-benzyl-5-phenyltetrazole P2X7 antagonists. J. Med. Chem. 49, 3659–3666. 10.1021/jm051202e 16759108

[B150] Ni J. Wang P. Zhang J. Chen W. Gu L. (2013). Silencing of the P2X(7) receptor enhances amyloid-beta phagocytosis by microglia. Biochem. Biophys. Res. Commun. 434, 363–369. 10.1016/j.bbrc.2013.03.079 23562658

[B151] Nicke A. (2008). Homotrimeric complexes are the dominant assembly state of native P2X7 subunits. Biochem. Biophys. Res. Commun. 377, 803–808. 10.1016/j.bbrc.2008.10.042 18938136

[B152] North R. A. (2002). Molecular physiology of P2X receptors. Physiol. Rev. 82, 1013–1067. 10.1152/physrev.00015.2002 12270951

[B153] North R. A. Jarvis M. F. (2013). P2X receptors as drug targets. Mol. Pharmacol. 83, 759–769. 10.1124/mol.112.083758 23253448 PMC3608433

[B154] Nv J.-C. I. (2017a). A Study to Investigate P2X7 Receptor Occupancy by JNJ-54175446 With the Newly Developed P2X7 Receptor Positron Emission Tomography (PET) Tracer 18F-JNJ-64413739 [Online]. Bethesda, MA: National Institues of Health.

[B155] Nv J.-C. I. (2017b). A Study to Investigate the Safety, Tolerability, and Pharmacokinetics of JNJ-55308942 in Healthy Male and Female Participants [Online]. National Institutes of Health. Available online at: https://clinicaltrials.gov/ct2/show/NCT03151486 (accessed October 10, 2020).

[B156] Oliveira-Giacomelli A. Naaldijk Y. Sarda-Arroyo L. Goncalves M. C. B. Correa-Velloso J. Pillat M. M.. (2018). Purinergic receptors in neurological diseases with motor symptoms: targets for therapy. Front. Pharmacol. 9:325. 10.3389/fphar.2018.00325 29692728 PMC5902708

[B157] Olla I. Santos-Galindo M. Elorza A. Lucas J. J. (2020). P2X7 receptor upregulation in Huntington's Disease Brains. Front. Mol. Neurosci 13, 567430. 10.3389/fnmol.2020.567430 33122998 PMC7573237

[B158] Ory D. Celen S. Gijsbers R. Van Den Haute C. Postnov A. Koole M.. (2016). Preclinical evaluation of a P2X7 receptor-selective radiotracer: PET studies in a rat model with local overexpression of the human P2X7 receptor and in nonhuman primates. J. Nucl. Med. 57, 1436–1441. 10.2967/jnumed.115.169995 27199364

[B159] Oyanguren-Desez O. Rodriguez-Antiguedad A. Villoslada P. Domercq M. Alberdi E. Matute C. (2011). Gain-of-function of P2X7 receptor gene variants in multiple sclerosis. Cell Calcium 50, 468–472. 10.1016/j.ceca.2011.08.002 21906809

[B160] Park J. H. Kim Y. C. (2017). P2X7 receptor antagonists: a patent review (2010-2015). Expert Opin. Ther. Pat. 27, 257–267. 10.1080/13543776.2017.1246538 27724045

[B161] Parvathenani L. K. Tertyshnikova S. Greco C. R. Roberts S. B. Robertson B. Posmantur R. (2003). P2X7 mediates superoxide production in primary microglia and is up-regulated in a transgenic mouse model of Alzheimer's disease. J. Biol. Chem. 278, 13309–13317. 10.1074/jbc.M209478200 12551918

[B162] Peng W. Cotrina M. L. Han X. Yu H. Bekar L. Blum L.. (2009). Systemic administration of an antagonist of the ATP-sensitive receptor P2X7 improves recovery after spinal cord injury. Proc. Natl. Acad. Sci. U.S.A. 106, 12489–12493. 10.1073/pnas.0902531106 19666625 PMC2718350

[B163] Pitkanen A. Lukasiuk K. (2011). Mechanisms of epileptogenesis and potential treatment targets. Lancet Neurol. 10, 173–186. 10.1016/S1474-4422(10)70310-0 21256455

[B164] Puchalowicz K. Baranowska-Bosiacka I. Dziedziejko V. Chlubek D. (2015). Purinergic signaling and the functioning of the nervous system cells. Cell. Mol. Biol. Lett. 20, 867–918. 10.1515/cmble-2015-0050 26618572

[B165] Pupovac A. Stokes L. Sluyter R. (2013). CAY10593 inhibits the human P2X7 receptor independently of phospholipase D1 stimulation. Purinergic Signal 9, 609–619. 10.1007/s11302-013-9371-6 23793974 PMC3889394

[B166] Ralevic V. Burnstock G. (1998). Receptors for purines and pyrimidines. Pharmacol. Rev. 50, 413–492. 9755289

[B167] Rees M. I. (2010). The genetics of epilepsy–the past, the present and future. Seizure 19, 680–683. 10.1016/j.seizure.2010.10.029 21094615

[B168] Ribeiro D. E. Roncalho A. L. Glaser T. Ulrich H. Wegener G. Joca S. (2019). P2X7 receptor signaling in stress and depression. Int. J. Mol. Sci. 20:2778. 10.3390/ijms20112778 31174279 PMC6600521

[B169] Rissiek B. Haag F. Boyer O. Koch-Nolte F. Adriouch S. (2015). P2X7 on mouse T cells: one channel, many functions. Front. Immunol. 6:204. 10.3389/fimmu.2015.00204 26042119 PMC4436801

[B170] Rodrigues R. J. Tome A. R. Cunha R. A. (2015). ATP as a multi-target danger signal in the brain. Front. Neurosci 9, 148. 10.3389/fnins.2015.00148 25972780 PMC4412015

[B171] Rodriguez-Alvarez N. Jimenez-Mateos E. M. Engel T. Quinlan S. Reschke C. R. Conroy R. M.. (2017). Effects of P2X7 receptor antagonists on hypoxia-induced neonatal seizures in mice. Neuropharmacology 116, 351–363. 10.1016/j.neuropharm.2017.01.005 28082183

[B172] Roszek K. Czarnecka J. (2015). Is ecto-nucleoside triphosphate diphosphohydrolase (NTPDase)-based therapy of central nervous system disorders possible? Mini-Rev. Med. Chem. 15, 5–20. 10.2174/1389557515666150219114416 25694082

[B173] Rudnick N. D. Griffey C. J. Guarnieri P. Gerbino V. Wang X. Piersaint J. A.. (2017). Distinct roles for motor neuron autophagy early and late in the SOD1(G93A) mouse model of ALS. Proc. Natl. Acad. Sci. U.S.A 114, E8294–E8303. 10.1073/pnas.1704294114 28904095 PMC5625902

[B174] Rudolph D. A. Alcazar J. Ameriks M. K. Anton A. B. Ao H. Bonaventure P.. (2015). Novel methyl substituted 1-(5,6-dihydro-[1,2,4]triazolo[4,3-a]pyrazin-7(8H)-yl)methanones are P2X7 antagonists. Bioorg. Med. Chem. Lett. 25, 3157–3163. 10.1016/j.bmcl.2015.06.004 26099534

[B175] Ruiz-Ruiz C. Calzaferri F. Garcia A. G. (2020). P2X7 receptor antagonism as a potential therapy in amyotrophic lateral sclerosis. Front. Mol. Neurosci. 13:93. 10.3389/fnmol.2020.00093 32595451 PMC7303288

[B176] Ryu J. K. Mclarnon J. G. (2008). Block of purinergic P2X(7) receptor is neuroprotective in an animal model of Alzheimer's disease. Neuroreport 19, 1715–1719. 10.1097/WNR.0b013e3283179333 18852683

[B177] Sadovnick A. D. Gu B. J. Traboulsee A. L. Bernales C. Q. Encarnacion M. Yee I. M.. (2017). Purinergic receptors P2RX4 and P2RX7 in familial multiple sclerosis. Hum. Mutat. 38, 736–744. 10.1002/humu.23218 28326637 PMC5429140

[B178] Sanz J. M. Chiozzi P. Ferrari D. Colaianna M. Idzko M. Falzoni S.. (2009). Activation of microglia by amyloid {beta} requires P2X7 receptor expression. J. Immunol. 182, 4378–4385. 10.4049/jimmunol.0803612 19299738

[B179] Savall B. M. Wu D. De Angelis M. Carruthers N. I. Ao H. Wang Q.. (2015). Synthesis, SAR, and pharmacological characterization of brain penetrant P2X7 receptor antagonists. ACS Med. Chem. Lett. 6, 671–676. 10.1021/acsmedchemlett.5b00089 26101572 PMC4468405

[B180] Schwarz N. Drouot L. Nicke A. Fliegert R. Boyer O. Guse A. H.. (2012). Alternative splicing of the N-terminal cytosolic and transmembrane domains of P2X7 controls gating of the ion channel by ADP-ribosylation. PLoS ONE 7:e41269. 10.1371/journal.pone.0041269 22848454 PMC3407210

[B181] Shemon A. N. Sluyter R. Conigrave A. D. Wiley J. S. (2004). Chelerythrine and other benzophenanthridine alkaloids block the human P2X7 receptor. Br. J. Pharmacol. 142, 1015–1019. 10.1038/sj.bjp.0705868 15210579 PMC1575114

[B182] Shemon A. N. Sluyter R. Fernando S. L. Clarke A. L. Dao-Ung L. P. Skarratt K. K.. (2006). A Thr357 to Ser polymorphism in homozygous and compound heterozygous subjects causes absent or reduced P2X7 function and impairs ATP-induced mycobacterial killing by macrophages. J. Biol. Chem. 281, 2079–2086. 10.1074/jbc.M507816200 16263709

[B183] Song P. Hu J. Liu X. Deng X. (2019). Increased expression of the P2X7 receptor in temporal lobe epilepsy: animal models and clinical evidence. Mol. Med. Rep 19, 5433–5439. 10.3892/mmr.2019.10202 31059094 PMC6522874

[B184] Sorge R. E. Trang T. Dorfman R. Smith S. B. Beggs S. Ritchie J.. (2012). Genetically determined P2X7 receptor pore formation regulates variability in chronic pain sensitivity. Nat. Med 18, 595–599. 10.1038/nm.2710 22447075 PMC3350463

[B185] Sperlagh B. Illes P. (2014). P2X7 receptor: an emerging target in central nervous system diseases. Trends Pharmacol. Sci. 35, 537–547. 10.1016/j.tips.2014.08.002 25223574

[B186] Sperlagh B. Kofalvi A. Deuchars J. Atkinson L. Milligan C. J. Buckley N. J.. (2002). Involvement of P2X7 receptors in the regulation of neurotransmitter release in the rat hippocampus. J. Neurochem. 81, 1196–1211. 10.1046/j.1471-4159.2002.00920.x 12068068

[B187] Sperlagh B. Vizi E. S. Wirkner K. Illes P. (2006). P2X7 receptors in the nervous system. Prog Neurobiol. 78, 327–346. 10.1016/j.pneurobio.2006.03.007 16697102

[B188] Stock T. C. Bloom B. J. Wei N. Ishaq S. Park W. Wang X.. (2012). Efficacy and safety of CE-224,535, an antagonist of P2X7 receptor, in treatment of patients with rheumatoid arthritis inadequately controlled by methotrexate. J. Rheumatol. 39, 720–727. 10.3899/jrheum.110874 22382341

[B189] Stokes L. Fuller S. J. Sluyter R. Skarratt K. K. Gu B. J. Wiley J. S. (2010). Two haplotypes of the P2X(7) receptor containing the Ala-348 to Thr polymorphism exhibit a gain-of-function effect and enhanced interleukin-1beta secretion. FASEB J. 24, 2916–2927. 10.1096/fj.09-150862 20360457

[B190] Stokes L. Jiang L. H. Alcaraz L. Bent J. Bowers K. Fagura M.. (2006). Characterization of a selective and potent antagonist of human P2X(7) receptors, AZ11645373. Br. J. Pharmacol. 149, 880–887. 10.1038/sj.bjp.0706933 17031385 PMC2014691

[B191] Sun C. Heid M. E. Keyel P. A. Salter R. D. (2013). The second transmembrane domain of P2X7 contributes to dilated pore formation. PLoS ONE 8:e61886. 10.1371/journal.pone.0061886 23613968 PMC3629090

[B192] Surprenant A. North R. A. (2009). Signaling at purinergic P2X receptors. Annu. Rev. Physiol. 71, 333–359. 10.1146/annurev.physiol.70.113006.100630 18851707

[B193] Surprenant A. Rassendren F. Kawashima E. North R. A. Buell G. (1996). The cytolytic P2Z receptor for extracellular ATP identified as a P2X receptor (P2X7). Science 272, 735–738. 10.1126/science.272.5262.735 8614837

[B194] Swanson D. M. Savall B. M. Coe K. J. Schoetens F. Koudriakova T. Skaptason J.. (2016). Identification of (R)-(2-Chloro-3-(trifluoromethyl)phenyl)(1-(5-fluoropyridin-2-yl)-4-methyl-6,7-di hydro-1H-imidazo[4,5-c]pyridin-5(4H)-yl)methanone (JNJ 54166060), a Small Molecule Antagonist of the P2X7 receptor. J. Med. Chem. 59, 8535–8548. 10.1021/acs.jmedchem.6b00989 27548392

[B195] Syed N. I. H. Kennedy C. (2012). Pharmacology of P2X receptors. Wiley Interdiscip. Rev. Membr. Transp. Signal. 1, 16–30. 10.1002/wmts.1

[B196] Territo P. R. Meyer J. A. Peters J. S. Riley A. A. Mccarthy B. P. Gao M.. (2017). Characterization of (11)C-GSK1482160 for targeting the P2X7 receptor as a biomarker for neuroinflammation. J. Nucl. Med. 58, 458–465. 10.2967/jnumed.116.181354 27765863

[B197] Tozaki-Saitoh H. Tsuda M. Inoue K. (2011). Role of purinergic receptors in CNS function and neuroprotection. Adv Pharmacol. 61, 495–528. 10.1016/B978-0-12-385526-8.00015-1 21586368

[B198] Van Weehaeghe D. Koole M. Schmidt M. E. Deman S. Jacobs A. H. Souche E.. (2019). [(11)C]JNJ54173717, a novel P2X7 receptor radioligand as marker for neuroinflammation: human biodistribution, dosimetry, brain kinetic modelling and quantification of brain P2X7 receptors in patients with Parkinson's disease and healthy volunteers. Eur. J. Nucl. Med. Mol. Imaging 46, 2051–2064. 10.1007/s00259-019-04369-6 31243495

[B199] Vazquez-Villoldo N. Domercq M. Martin A. Llop J. Gomez-Vallejo V. Matute C. (2014). P2X4 receptors control the fate and survival of activated microglia. Glia 62, 171–184. 10.1002/glia.22596 24254916

[B200] Volonte C. Amadio S. Liguori F. Fabbrizio P. (2020). Duality of P2X7 receptor in amyotrophic lateral sclerosis. Front. Pharmacol. 11:1148. 10.3389/fphar.2020.01148 32792962 PMC7394054

[B201] Volonte C. Apolloni S. Parisi C. Amadio S. (2016). Purinergic contribution to amyotrophic lateral sclerosis. Neuropharmacology 104, 180–193. 10.1016/j.neuropharm.2015.10.026 26514402

[B202] Volonte C. Apolloni S. Skaper S. D. Burnstock G. (2012). P2X7 receptors: channels, pores and more. CNS Neurol. Disord. Drug Targets 11, 705–721. 10.2174/187152712803581137 22963440

[B203] Vuorimaa A. Rissanen E. Airas L. (2017). *In Vivo* PET imaging of adenosine 2A receptors in neuroinflammatory and neurodegenerative disease. Contrast Media Mol. Imaging 2017:6975841. 10.1155/2017/6975841 29348737 PMC5733838

[B204] Wang X. Arcuino G. Takano T. Lin J. Peng W. G. Wan P.. (2004). P2X7 receptor inhibition improves recovery after spinal cord injury. Nat. Med 10, 821–827. 10.1038/nm1082 15258577

[B205] Wang X. H. Xie X. Luo X. G. Shang H. He Z. Y. (2017). Inhibiting purinergic P2X7 receptors with the antagonist brilliant blue G is neuroprotective in an intranigral lipopolysaccharide animal model of Parkinson's disease. Mol. Med. Rep 15, 768–776. 10.3892/mmr.2016.6070 28035410 PMC5364844

[B206] Watch C. (2021). A Study to Investigate the Safety Tolerability and Pharmacokinetics of JNJ-55308942 in Healthy Male and Female Participants [Online]. Center Watch. Available online at: https://www.centerwatch.com/clinical-trials/listings/118525/healthy-study-investigate-safety-/ (accessed May 10, 2020).

[B207] Weisman G. A. Camden J. M. Peterson T. S. Ajit D. Woods L. T. Erb L. (2012). P2 receptors for extracellular nucleotides in the central nervous system: role of P2X7 and P2Y(2) receptor interactions in neuroinflammation. Mol. Neurobiol. 46, 96–113. 10.1007/s12035-012-8263-z 22467178 PMC3439567

[B208] Wiley J. S. Dao-Ung L. P. Li C. Shemon A. N. Gu B. J. Smart M. L.. (2003). An Ile-568 to Asn polymorphism prevents normal trafficking and function of the human P2X7 receptor. J. Biol. Chem. 278, 17108–17113. 10.1074/jbc.M212759200 12586825

[B209] Wiley J. S. Sluyter R. Gu B. J. Stokes L. Fuller S. J. (2011). The human P2X7 receptor and its role in innate immunity. Tissue Antigens 78, 321–332. 10.1111/j.1399-0039.2011.01780.x 21988719

[B210] Wilkaniec A. Gassowska M. Czapski G. A. Cieslik M. Sulkowski G. Adamczyk A. (2017). P2X7 receptor-pannexin 1 interaction mediates extracellular alpha-synuclein-induced ATP release in neuroblastoma SH-SY5Y cells. Purinergic Signal 13, 347–361. 10.1007/s11302-017-9567-2 28516276 PMC5563296

[B211] Yang H. S. Onos K. D. Choi K. Keezer K. J. Skelly D. A. Carter G. W.. (2021). Natural genetic variation determines microglia heterogeneity in wild-derived mouse models of Alzheimer's disease. Cell Rep 34:108739. 10.1016/j.celrep.2021.108739 33567283 PMC7937391

[B212] Yegutkin G. G. (2008). Nucleotide- and nucleoside-converting ectoenzymes: Important modulators of purinergic signalling cascade. Biochim. Biophys. Acta 1783, 673–694. 10.1016/j.bbamcr.2008.01.024 18302942

[B213] Yiangou Y. Facer P. Durrenberger P. Chessell I. P. Naylor A. Bountra C.. (2006). COX-2, CB2 and P2X7-immunoreactivities are increased in activated microglial cells/macrophages of multiple sclerosis and amyotrophic lateral sclerosis spinal cord. BMC Neurol. 6, 12. 10.1186/1471-2377-6-12 16512913 PMC1413551

[B214] Yoshida K. Ito M. Matsuoka I. (2015). P2X7 receptor antagonist activity of the anti-allergic agent oxatomide. Eur. J. Pharmacol. 767, 41–51. 10.1016/j.ejphar.2015.10.002 26463039

[B215] Yu Q. Guo Z. Liu X. Ouyang Q. He C. Burnstock G.. (2013). Block of P2X7 receptors could partly reverse the delayed neuronal death in area CA1 of the hippocampus after transient global cerebral ischemia. Purinergic Signal 9, 663–675. 10.1007/s11302-013-9379-y 23877788 PMC3889395

[B216] Yue N. Huang H. Zhu X. Han Q. Wang Y. Li B.. (2017). Activation of P2X7 receptor and NLRP3 inflammasome assembly in hippocampal glial cells mediates chronic stress-induced depressive-like behaviors. J. Neuroinflamm. 14:102. 10.1186/s12974-017-0865-y 28486969 PMC5424302

[B217] Zarrinmayeh H. Territo P. R. (2020). Purinergic receptors of the central nervous system: biology, PET ligands, and their applications. Mol. Imaging 19:1536012120927609. 10.1177/1536012120927609 32539522 PMC7297484

[B218] Zhang K. Liu J. You X. Kong P. Song Y. Cao L.. (2016). P2X7 as a new target for chrysophanol to treat lipopolysaccharide-induced depression in mice. Neurosci. Lett. 613, 60–65. 10.1016/j.neulet.2015.12.043 26724370

[B219] Zheng J. Jiang Y. Y. Xu L. C. Ma L. Y. Liu F. Y. Cui S.. (2017). Adult hippocampal neurogenesis along the dorsoventral axis contributes differentially to environmental enrichment combined with voluntary exercise in alleviating chronic inflammatory pain in mice. J. Neurosci. 37, 4145–4157. 10.1523/JNEUROSCI.3333-16.2017 28292830 PMC6596585

[B220] Zhu B. Lu Y. Chen L. Yu B. Liu Y. Song M.. (2017). Identification and characterization of related substances in EVT-401 by hyphenated LC-MS techniques. J. Pharm. Anal. 7, 223–230. 10.1016/j.jpha.2017.03.008 29404042 PMC5790700

